# Weighted Ensemble Simulations Reveal Novel Conformations and Modulator Effects in Hepatitis B Virus Capsid Assembly

**DOI:** 10.1021/acs.jctc.5c01197

**Published:** 2025-11-25

**Authors:** Diane L. Lynch, Anna Pavlova, Zixing Fan, James C. Gumbart

**Affiliations:** † School of Physics, 1372 Georgia Institute of Technology, Atlanta, Georgia 30332, United States; ‡ Interdisciplinary Bioengineering Graduate Program, 1372 Georgia Institute of Technology, Atlanta, Georgia 30332, United States; § School of Chemistry & Biochemistry, 1372 Georgia Institute of Technology, Atlanta, Georgia 30332, United States

## Abstract

Molecular dynamics (MD) simulations provide a detailed description of biophysical processes, allowing mechanistic questions to be addressed at the atomic level. The promise of such approaches is partly hampered by well-known sampling issues of typical simulations, where time scales available are significantly shorter than the process of interest. For the process of interest here, the binding of modulators of Hepatitis B virus capsid self-assembly, the binding site is at a flexible protein–protein interface. Characterization of the conformational landscape and how it is altered upon ligand binding is thus a prerequisite for a complete mechanistic description of capsid assembly modulation. However, such a description can be difficult due to the aforementioned sampling issues of standard MD, and enhanced sampling strategies are required. Here, we employ the weighted ensemble methodology to characterize the free-energy landscape of our earlier determined functionally relevant progress coordinates. It is shown that this approach provides conformations outside those sampled by standard MD, as well as an increased number of structures with correspondingly enlarged binding pockets conducive to ligand binding, illustrating the utility of weighted ensemble for computational drug development.

## Graphical abstract

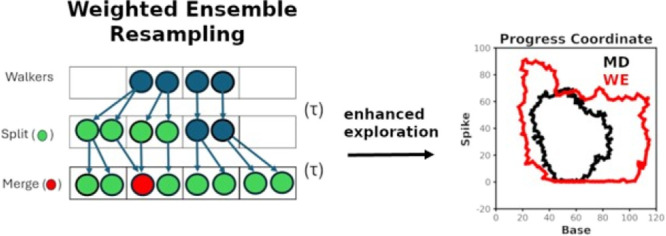

## Introduction

Biological function is determined not only by protein structure but also dynamics; as such characterizing the thermally accessible conformational landscape is essential for understanding the full range of functional outcomes. Significant progress has been made in experimental approaches to resolve the underlying dynamics for proteins, for example solid-state NMR,^1^ HDX-MS,^2^ and time-resolved cryo-EM^3^ among others; however, transient states often remain experimentally inaccessible. While MD simulations in principle can fully describe the atomistic dynamics, and significant progress has been made in the application of molecular simulations to viral systems,^4^ typical all-atom MD often can not reach physiologically relevant time scales. For example, it is only with specialized hardware that Ayaz et al.^5^ observe both ligand binding and resulting conformational changes from MD simulations employing hundreds of μs of simulation.

Chronic Hepatitis B virus (HBV) infection leads to severe liver damage and is the leading cause of liver disease^6^ with approximately a million deaths per year.^7^ HBV is an enveloped nucleocapsid virus, including an icosahedral shell structure comprising 120 core protein (Cp) homodimers (Figure 1) containing the viral genome, which is surrounded by a membrane envelope.^8^ The capsid contains, protects, and delivers the viral genome during the HBV life cycle. Recent work has shown that a promising approach for reducing viral replication is to target the HBV nucleocapsid with ligands that disrupt capsid self-assembly (capsid assembly modulators or CAMs) producing noninfectious particles.^9,10^ The continued development of CAMs is an active area of research.^7,9–13^

**Figure 1:**
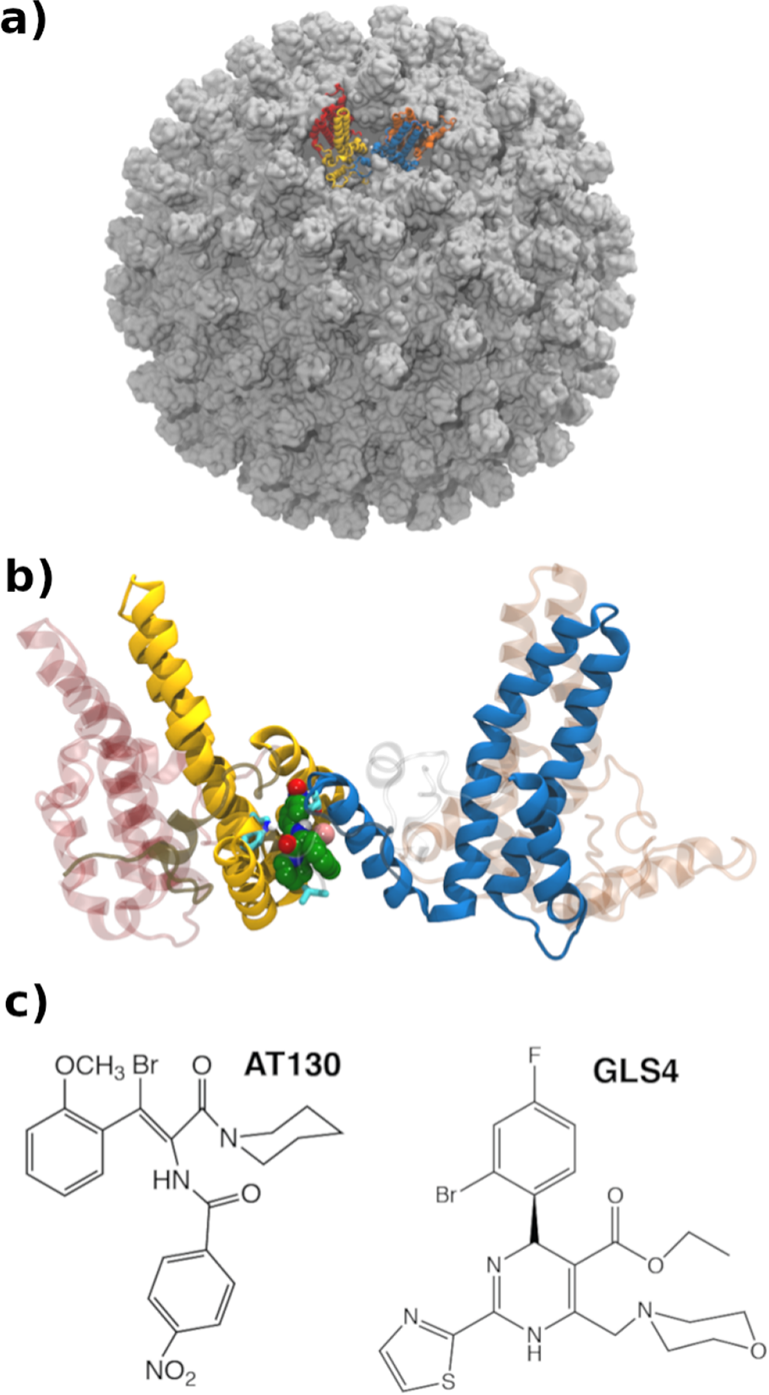
HBV capsid structure (PDB: 6HU4). (a) 240-unit capsid structure contains four quasi-equivalent tetramers. The ABCD tetramer, used for earlier MD studies,^17^ is rendered in ribbon with the A, B, C, and D subunits colored in red, gold, blue, and dark orange, respectively. (b) Tetrameric unit with a bound CAM shown in a space-filling representation. (c) Chemical structures of AT-130 and GLS4. Structures rendered using PDB 6HU4.

The building blocks of HBV capsids are Cp dimers, which oligomerize into early assembly intermediates, e.g., tetramers and hexamers, and ultimately the full capsid.^14,15^ These earlier studies,^14,15^ supported by MD simulations,^16,17^ posit that the dimer’s conformation must undergo a shift to an “assembly active” state for productive assembly. As such, capsid formation is described by a conformational selection mechanism and is governed to a large part by the underlying conformational landscape of early assembly intermediates. Reshaping this conformational landscape by ligand binding (e.g., CAMs) leads to altered assembly pathways and ultimately the production of defective, noninfectious viral particles.

Currently CAMs are distinguished based on their effects on capsid formation, where class II compounds are those that accelerate the production of normal, yet empty capsids^18,19^ (e.g., phenylpropenamides), while class I CAMs generate aberrant structures such as tubes and sheets^20,21^ (e.g., heteroaryldihydropyrimidines). Examples of class I and class II compounds include GLS4 and AT-130, respectively (Figure 1c). Although assembly outcomes are significantly different, surprisingly CAMs share the same binding site at the tetrameric interdimer interface (Figure 1b). Mechanistically this points to allosteric effects,^22^ with CAM-specific alterations in the tetramer conformational ensemble, although rational control of such effects remain challenging.

Given the allosteric nature of HBV capsid assembly modulation, a detailed mechanistic description for capsid assembly will require a comprehensive evaluation of CAM-specific modulations of the conformational ensemble. Moreover, comprehensive docking efforts in structure-based drug design programs commonly employ ensemble docking, where protein flexibility is approximated by using a collection of diverse protein structures.^23^ However, the success of such endeavors is dependent on the structures comprising these collections,^24^ which can be limited in the conformational space explored in standard MD simulations.

Various approaches to more fully explore the free energy landscape have been employed in MD applications, such as metadynamics, replica-exchange MD, or other enhanced sampling approaches.^25^ In addition, a straightforward approach to accelerate sampling is to employ hydrogen mass repartitioning (HMR),^26^ allowing a larger (4 fs) time step for MD simulations. Earlier reports have established the validity of HMR in the equilibrium sampling of proteins^26^ as well as membrane systems.^27^ Although Jung et al.^28^ and Votapka et al.^29^ report that the application of HMR reproduces both thermodynamic and kinetic properties for several systems, including protein–ligand systems, more recently, the use of HMR for protein–ligand kinetic quantities has been questioned.^30^ Intriguingly, the weighted ensemble (WE) simulation approach^31^ has been developed for rare-event processes and rigorously describes kinetics, although it has also been employed to effectively generate an equilibrium ensemble.^32,33^ WE simulations offer the benefit of both enhanced sampling as well as unbiased dynamics and has been applied to a variety of systems, including *k*_on_ and *k*_off_ rates of protein–protein or protein–ligand binding,^34–36^ protein folding events,^37^ conformational changes,^38–40^ drug-membrane partitioning,^41^ as well as phase separation^42^ and continues to be developed with an open source python-based implementation readily available.^43,44^ Moreover, Xu et al.^45^ and Hellemann and Durrant^46^ have recently applied WE simulations for the study of ligand binding site properties in the context of ensemble docking studies.

Here, we have employed WE simulations, coupled with HMR, to explore the conformational space of apo, as well as CAM-bound, HBV tetramers. We demonstrate that WE-generated apo structures are capable of binding both class I (GLS4) as well as class II (AT-130) HBV CAM ligands, with an increase in the number of conformations with large-volume ligand binding sites. Moreover, given the recent report of difficulties of HMR with ligand-based systems,^30^ we carry out a comparison of standard time-step MD with HMR for apo and CAM-bound (AT-130 and GLS4; Figure 1c) tetrameric systems. Overall, we find that the use of HMR in equilibrium simulations does not significantly perturb these protein–ligand systems and that WE simulations provide an effective approach for producing an expanded representation of the conformational ensemble, including a collection of unique structures that are difficult to access via standard MD.

## Methods

### Model Construction and MD Propagation Parameters

The starting structures for the apo, AT-130, and GLS4 simulations, based on the 3J2V,^47^ 4G93,^48^ and 5E0I^49^ (where the bound ligand was changed to GLS4) structures, respectively, were taken from the previous equilibrated simulations of Pavlova et al.^17^ and minimized for 2000 steps prior to simulation. All MD simulations were carried out with NAMD3 ^50^ using the CHARMM36m force field for proteins^51^ along with the TIP3P water model.^52^ CGenFF^53^ parameters for GLS4 were taken from Pavlova et al.,^17^ while parameters for AT-130 were taken from Pang et al.^54^ In all simulations, the van der Waals cutoff was set to 12 Å, with a smoothing function applied from 10 to 12 Å. Long-range electrostatics were calculated using the particle mesh Ewald method.^55^ A constant temperature of 310 K was maintained using Langevin dynamics and a constant pressure of 1 atm was maintained using the Langevin piston method available in NAMD.^50^ Specifically, the Langevin thermostat, using a damping coefficient of 1 ps^–1^ and the Langevin piston^56^ with a period of 200 fs and a decay of 100 fs were used to control the temperature and the pressure, respectively.

For standard molecular dynamics, each system was run for 500 ns employing 12 replicas for an aggregate of 6 μs of simulation. For these standard MD simulations, either a 2 fs or an HMR scheme was employed, with the latter allowing the use of a 4 fs time step.^26,27^ Importantly, each replica in the apo, GLS4, and AT-130 systems, regardless of time step, was initiated from a common initial configuration, taken from a short equilibration run from the minimized structure.

MD trajectory propagation settings for the evolution of the WE simulations were identical to those of the standard HMR MD described above. The initial configuration was taken after ∼40 ns of standard MD from the first HMR replica described above. Two independent WE replicas were simulated, each starting from this same initial configuration. WE specific parameters are described further below.

### Weighted Ensemble

In the current WE simulations a 2-D progress coordinate was chosen based on the results of earlier work,^17^ which identified specific dimer–dimer orientations of the HBV tetramer, described by the base and spike angles (Figure 2a), sensitive to the presence and/or type of CAM. The base angle reflects an opening or closing of the Cp dimers in the tetramer and is calculated based on the geometry of the constituent α5 helices, defined using the backbone atoms of residues 111 to 127. Specifically, for each dimer the vector between the geometric centers of the two α5 helices is computed, and the angle between these two intradimer vectors is the base angle. The geometry of the α3 and α4 helices is used in the spike angle definition. Dividing these helices into an upper and lower portion about a flexible hinge generates two segments: an upper segment using residues 49 to 56 and residues 103 to 110 and a lower segment using residues 56 to 65 and 96 to 103. Given this definition, the vector from the lower to upper center is evaluated for each dimer, and the angle between these resultant vectors is the spike angle.

**Figure 2:**
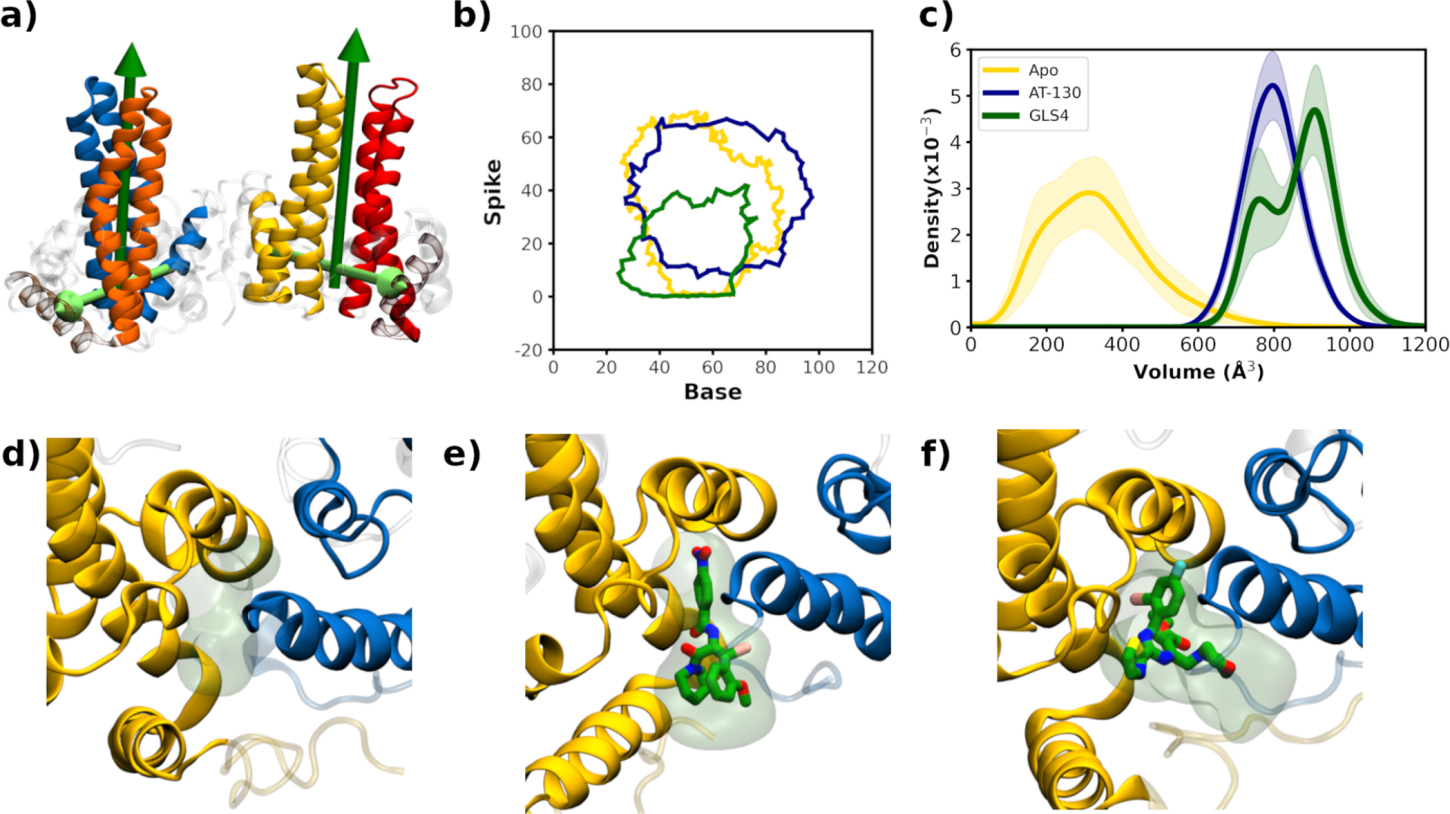
Results of standard MD simulations. (a) Illustration of the base and spike angles, with the vectors describing the base/spike angles rendered a light green/dark green, respectively, while the tetramer is displayed as described in Figure 1b. (b) Base and spike angles for the apo (gold), AT-130 (dark blue), and GLS4 (dark green) rendered as a boundary from our standard MD simulations. (c) Distribution of binding pocket volumes computed with Epock for the apo, AT-130-bound, and GLS4-bound standard MD simulations with 95% confidence intervals added as shaded regions. (d–f) Structures of the binding pocket volumes for d) apo (volume = 287 Å^3^), (e) AT-130 (volume = 785 Å^3^), and (f) GLS4 (volume = 955 Å^3^). Chain B is rendered in gold ribbon, chain C in dark blue ribbon, the ligand is rendered in licorice with the carbons, oxygen, sulfur, fluorine, or bromine atoms green, red, yellow, cyan, or pink. The Epock-derived volume is a transparent green surface.

Although WE simulations are often run in order to provide unbiased kinetic data using steady-state conditions, equilibrium conditions can also be applied. In fact WE has been used in order to effectively sample such equilibrium populations.^32,34^ Analogous to the initial/preparatory equilibrium simulations for peptide–protein and protein–protein interactions^33,37^ we employed equilibrium WE simulation using WESTPA version 1 ^57^ (release 2020.06) with 2-D progress coordinates specified by the base and spike angles discussed above until reasonable convergence was obtained for the larger population regions. The simulations were performed using equilibrium conditions, with a τ = 50 ps, 8 trajectories per bin, and the bins subdivided at 20° boundaries for both the base and spike angles, initially set at [15,35,55,75,95] and [10,30,50,70,90] respectively. Given the variance observed in multiple WE runs,^37^ and as recommended,^58^ two independent WE simulations were performed for ∼3 μs each. In the case of the apo simulations this resulted in 650/960 iterations for each replica respectively, while for AT-130 and GLS4-bound simulations the iterations per replica are 610/500 and 1350/1365 respectively. Note, when the progress coordinate exceeded the extreme bin boundaries these were subsequently extended. The evolution of each progress coordinate and the 2-D landscape for each replica were generated using the WESTPA tools *w_pdist* and *plothist*, with the replicas combined using *w_multi_west*.

### Analysis

Unless otherwise noted, visualization and analysis were done using VMD version 1.9.4.^59^ Pairwise root-mean-square deviation (RMSD) calculations were performed with MDTraj,^60^ while Delaunay triangulation, available in SciPy,^61^ was used for generating progress coordinate boundaries. Distributions were generated using the seaborn package^62^ and applying a Gaussian kernel estimate. Matplotlib^63^ version 3.3.4 was used for general plotting and histogram analysis.

#### Epock Analysis

CAM pocket volumes were computed using Epock.^64^ The pocket was identified as those residues making a close contact (4.5 Å) between heavy atoms of the ligand in the AT-130 and GLS4 systems and include residues 121, 124, 125, 127, 128, 129, 132, 133, 134, 136, and 138 from chain C and residues 23, 25, 29, 30, 33, 37, 102, 105, 106, 109, 110, 114, 115, 118, 138, 139, and 140 from chain B. The volume was obtained using a spherical probe (10-Å radii) along with noncontiguous points removal, which removes free space points outside a given radius (here taken as 3 Å). Both are centered on the geometrical center of the binding site residues listed above. Additionally an exclusion region, defined by a 3-Å sphere centered at the COM of the side chains of chain B residue 29 and chain C residues 127, 129, and 133 was used. Binding pocket volumes using these choices are illustrated in Figure S1, where ligands under study are effectively encompassed.

#### Clustering Analysis

The structures used for clustering were taken from the collection of WE (unweighted) or standard MD conformations; the number of conformations used was ∼51,000 and 54,000 for the WE and standard MD, respectively, in order to cluster approximately the same amount of data. This amounts to all the available standard MD conformations, with a similar frequency chosen in the WE analysis for comparative purposes. The difference in the total conformations primarily results from differences in the WE algorithm, which generates data after each iteration. In cases where there is significant conformational alteration of the binding site due to ligand-induced steric effects,^65^ such conformations may appear with low probability in apo simulations, yet these conformations become highly desirable in ensemble docking methods.^23^ This approach is particularly advantageous in cases where holo structures are unavailable. Therefore, we used the unweighted data to avoid missing conformations that are rare in apo simulations yet are relevant to ligand binding of CAMs, i.e., have significantly shifted base and spike angles relative to what is observed in apo simulations. In both cases, initial data was dropped prior to analysis: 10 ns in the case of standard MD and where the highly populated bin weights were approximately leveled off for the WE simulations, at 250 iterations. Xu et al.^45^ employed a different approach, with the WE simulations using ligand binding site information (solvent-accessible surface area) to sample alternate conformations, and with the clustering performed using a representative pathway taken from the WE simulation. Moreover, clustering WE simulations has been discussed by Hellemann and Durrant,^46^ where they employed a two-tier approach to avoid striding over the branching points in a WE simulation.

In the present case, we have focused on the functionally important motion associated with HBV capsid assembly modulation,^13,17^ i.e., CAM-mediated shifting of the base and spike angles, which are not necessarily sampled in standard apo simulations. The clustering of the base and spike angles was performed with SciPy^61^ hierarchical agglomerative clustering using average linkage and a precomputed distance matrix. The distance matrix employed here is constructed using the minimum arc length of the 2-D angular coordinates, with the distance itself calculated as the euclidean norm of these circular variables, metrics that are commonly used when treating circular data.^66,67^ We reasoned that to ensure that the structures chosen for docking spanned the relevant conformational space in this system, a straightforward approach is to partition the base/spike conformational space itself. Thus, we clustered directly on the base/spike pair. In general, few studies have been made that directly compare clustering methods/features; for example Gan et al.^68^ highlight that the choice of features remains challenging and often is based on detailed knowledge of the specific system.

#### Docking

AT-130 and GLS4 were docked into the CAM binding pocket of the target protein using AutoDock Vina (version 1.1.2).^69,70^ To account for potential conformational adjustments during docking, the side chains of residues within a 6-Å radius of the ligands were set as flexible. This flexibility ensures accurate modeling of protein–ligand interactions in the binding pocket.

The docking simulation explored a search space defined as a 30 Å × 30 Å × 30 Å box, centered on the original position of AT-130 and GLS4 within their bound conformations (PDB codes 4G93 and 5E0I, respectively). The center of this box was determined after structural alignment of the protein conformations to the GLS4-bound structure. Docking was performed using an exhaustiveness value of 32 to increase search thoroughness and promote result reproducibility.

## Results and Discussion

### HMR Produces Only Modest Effects on the Conformational Sampling of the HBV Tetramer

In the course of our work, Sahil et al.^30^ reported sensitivity of ligand unbinding kinetics to the use of HMR. Their study illustrated that the use of HMR produces slowed ligand binding events, attributed to the effects of the longer time step on both ligand diffusion, an effect observed earlier,^27^ and protein fluctuations. Here we have studied apo, AT-130-, and GLS4-bound HBV tetramers using both a standard 2 fs time step as well as HMR (4 fs time step) in order to characterize the distributions observed and point to any potential artifacts introduced via the application of the longer time step. Using 12 500-ns replicas for each system, RMSD to the starting configuration, as well as the pairwise (all to all) RMSDs, which characterize the conformational space explored, were computed. In addition, after protein alignment, CAM RMSDs, using the ligand heavy atoms, were computed.

On average, the 2 fs and HMR results display similar behavior for the systems studied, i.e., apo, AT-130-bound, and GLS4-bound. The average RMSD computed using the 2 and 4 fs trajectories compared to the starting structure for protein, CAM binding pocket, and ligand are within standard error (Figure S2). Although the RMSD distributions for individual replicas appear noisy (Figure S3), the overall protein distributions are quite similar (Figure S3a), while the analogous distributions for the CAM binding site using the 4 fs data appear shifted and somewhat wider than the 2 fs results (Figure S3d). Pairwise RMSDs (Figures S4 and S5) for the protein and CAM binding site were also computed, with the pairwise computation measuring RMSD over the entire set of frames, rather than deviation from just the initial structure. Again, although the individual replicas appear noisy, the overall protein distributions are quite similar (Figure S5a), while the CAM binding pocket in the 4 fs trajectories appears variable and slightly shifted (Figure S5d–f). Analogous analysis was performed for the ligand bound AT-130 and GLS4 simulations (Figures S6–S11) with similar results to those observed for the apo simulations, i.e., the introduction of HMR produces very small alterations in the protein structures, while the CAM binding site appears more susceptible to noise. However, it is important to note that convergence of such simulations can be difficult, with multiple replicas recommended with the number dependent on the length of the trajectory as well as the system under study.^71^ Given the goal in the present study is the exploration of the equilibrium conformational ensemble, rather than kinetic properties^30^ of the HBV tetramer, we have used HMR in the following WE simulations.

### Effects of CAM Binding on Tetrameric Structures

Our biophysics-based approach^13,17^ for exploring the molecular details of CAM-specific capsid assembly modulation has revealed CAM-class specific altered tetrameric conformations, which are associated with accelerated, yet normal (Class II), or aberrant capsid (Class I) assembly. Upon CAM binding, ligand-specific structural changes of the HBV dimer occur, which generate intermediates that ultimately lead to altered capsid assembly. As such CAM binding is characterized by both its binding affinity as well as the degree to which it alters the underlying tetrameric conformational ensemble.

Pavlova et al.^17^ established that angles defining the interdimer orientation, i.e., the base and spike angles (Figure 2a), are effective at discriminating the conformational preferences of the apo, as well as CAM-bound, tetramers. The base and spike angles describe the opening and closing or bending of the tetramer respectively, with the base angle defined by the α5 helices of the Cp monomers and the spike angle defined by the relative direction of the α3/α4 helices in each dimer. From these earlier simulations it was apparent that the combination of these two angles characterized the observed ligand-dependent conformational variation in apo and CAM-bound simulations.^13,17^ Relative to apo, binding of class I (misdirectors) or class II (accelerators) shifts the base and spike angles to smaller or larger values, respectively. Importantly these angles provide a mechanistically relevant reduced representation for describing the CAM-specific modulation of the dynamics of the tetrameric interdimer orientations.

### Standard MD Simulations of the HBV Tetramer Produce Limited Exploration of Base and Spike Angles

The HMR-based standard MD replicas described above, whose RMSD have all nearly reached a plateau (Figure S13), were used to evaluate the apo and CAM-specific base and spike angle landscape (Figure 2b), with the angle data represented by a boundary encompassing all the data points. The class I misdirector (GLS4) base and spike angle pair are shifted to lower values and explore a more restricted region of interdimer orientations relative to those of the apo and AT-130 simulations, particularly the spike angle (average values and standard errors are reported in Figure S12). Similar results for the base and spike angles were observed in our previous work.^17^ The apo and AT-130 results are clearly similar, and although noisy, the AT-130 simulations reach larger base/spike values. As detailed in Pavlova et al.,^17^ this shift to larger angles increases the conformational overlap with Cp hexamers, which has been associated with capsid assembly acceleration, thereby reflecting the mechanism of action of AT-130.

The binding site for CAM compounds, regardless of functional outcome, lies at the interface of the B and C chains in the tetrameric unit of the capsid (Figure 1b). We have evaluated the CAM-binding-site volumes (see Methods for residues used) from the apo, AT-130, and GLS4 simulations (illustrated in Figure 2d–f) using Epock.^64^ The distributions observed in the apo simulations are spread over a large range and reflect the flexibility of the apo binding site (Figure 2c), while the pocket is well-defined in the AT-130- and GLS4-bound simulations. Relative to AT-130, the GLS4 ligand displays a somewhat larger available volume. The secondary maximum at lower volume in the GLS4 simulations corresponds to a set of structures where the C-terminus extends around the ligand limiting available space (see Figure S14 for an illustration). Of particular note, very few structures from the apo simulation could accommodate either ligand, highlighting a known issue with using structures from apo simulations for docking.^65^

### WE Simulations More Effectively Explore Base and Spike Angles

WE is a resampling strategy designed to produce rare-event transitions, without the addition of applied forces, along an appropriately chosen progress coordinate or coordinates^31^ and belongs to the path-sampling class of enhanced sampling approaches. In addition to steady-state conditions, where an initial state is transitioned to a predefined target state, equilibrium conditions are also possible.^32,72^ An advantage of the latter is that a target state definition is not necessary; as such, these simulations explore the conformational space of the system in an unbiased fashion.

Two replicas of WE simulations for the apo HBV tetramers were performed (6 μs total) employing 2-D progress coordinates consisting of the base and spike angles discussed above. As detailed in Methods, each angle was partitioned into 20° bins. The evolution of the base and spike angles are provided in Figure S15, while the 2-D landscape generated from the combination of the two replicas is illustrated in Figure 3a. It is apparent that although a single structure was employed to start the WE simulations, the equilibrium distribution spreads out such that a wide range of angles are sampled. In Figure 3b,c, the weights as a function of iteration for two highly populated bins for the individual replicas are reported. Similar to the distribution of rate constants reported by Adhikari et al.,^37^ each replica behaves somewhat differently, with Replica 2 showing a narrower exploration of the base/spike progress coordinates (Figure S15b). Given this slight dependence on WE replica, we ran Replica 2 for an additional 200 iterations (Figure S15c) to illustrate that the expanded WE exploration of base and spike angles is reproducible. Although reasonable convergence of the highly populated bins is achieved (Figure 3b,c), even for runs of 3 μs, convergence of the smaller weights appears more problematic, e.g., the magnitude of the smaller bin weights oscillates dramatically with small changes in population (Figure S16).

**Figure 3:**
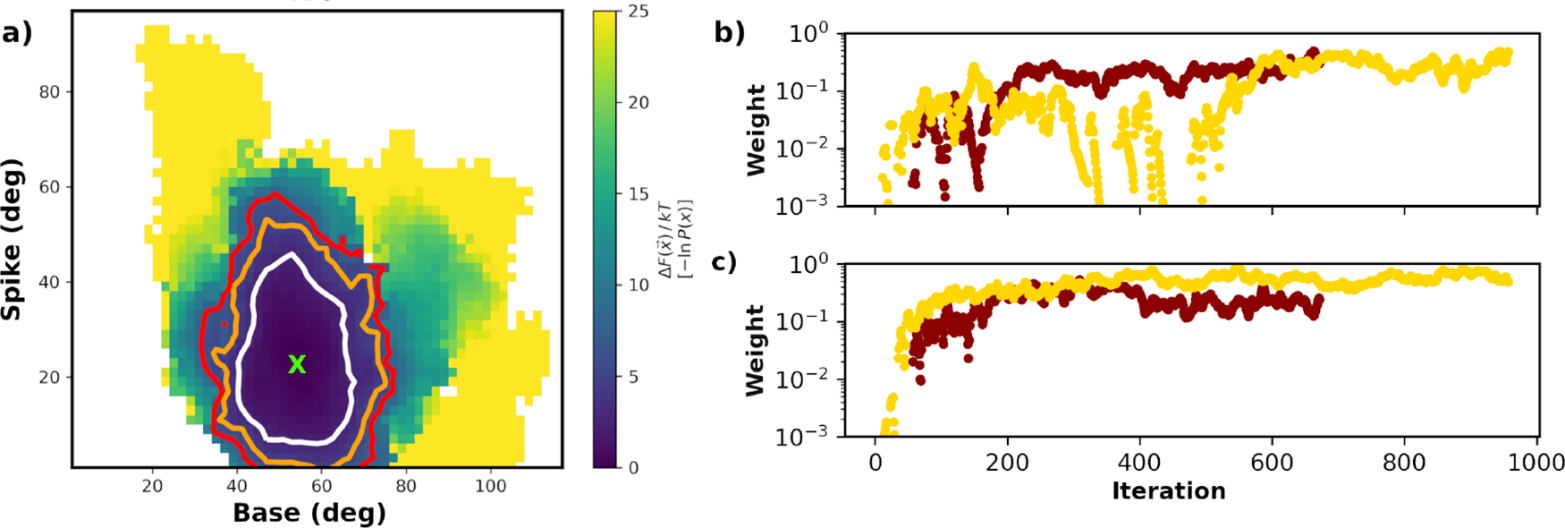
Apo WE simulations. (a) 2-D free energy landscape after combining the two replicas. Contoured at 2.5*kT* (white), 5.0*kT* (orange), and 7.5*kT* (red) and removing the first 250 iterations. The initial base/spike angle pair is indicated with a green X. (b,c) Bin weights as a function of WE iteration for bin 6 (base angles = 35°–55°, spike angles = 10°–30°) and bin 7 (base = 35°–55°, spike = 30°–50°), with replica 1 or 2 colored dark red or gold, respectively.

Analogous WE simulations, two replicas of 3 μs each, were performed for AT-130- and GLS4-bound tetramers, and, similar to the apo results, the major bins appear reasonably well converged. Figures S17–S20 display progress coordinate evolution, the 2-D landscapes, and the weights as a function of iteration for these simulations. The resulting combined 2-D landscapes for the CAM-bound simulations (Figure 4) and progress coordinate evolution (Figures S17 and S19) reveal an altered exploration pattern for the GLS4-bound simulations relative to those for the apo and AT-130-bound systems. Similar to the apo WE results, the AT-130-bound tetramer explores a wide range of base and spike angles and is consistent with the shifting of these angles to larger values relative to the apo simulations (Figure 3a) as observed in standard MD (Figure 2b). Despite the fact that the current WE settings provide robust sampling along the base/spike progress coordinates for both apo and AT-130 simulations, in the case of GLS4, both replicas explore a much narrower range (Figures 4b and S19). Although these results are consistent with the observations that GLS4, a misdirector, does not readily sample the interdimer orientations necessary for normal capsid assembly,^13,17^ it is possible that a more aggressive rebinning during the WE simulations could provide further exploration of these interdimer orientations. As recently reviewed by Chong and Zuckerman,^44^ the WE approach is under active development. Adaptive binning,^73^ which focuses the simulation on poorly explored regions of progress coordinate space, improves the efficiency of sampling these regions. Moreover, recently machine-learning (ML)^74,75^ and deep-learning^76^ extensions have been shown to assist in the refinement of the progress coordinates themselves. We have recently demonstrated that ML classification tasks can reveal subtle conformational differences between apo and various CAM-bound HBV tetramers.^77^ These novel approaches offer the promise of improving the sampling of the conformational landscape of difficult systems, such as GLS4-bound HBV tetramers.

**Figure 4:**
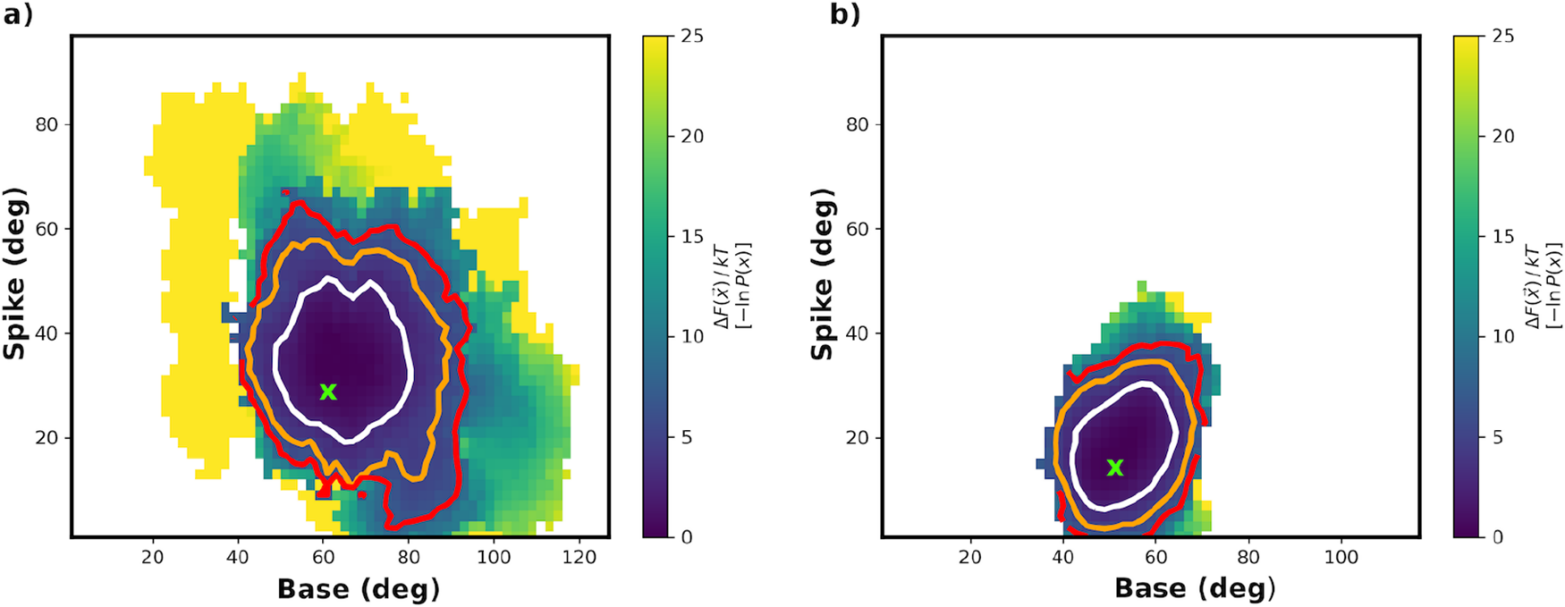
AT-130 and GLS4 WE 2-D energy landscapes. (a) AT-130 and (b) GLS4 results after combining the two replicas (3 μs for each replica). Contoured at 2.5*kT* (white), 5.0*kT* (orange), and 7.5*kT* (red) and removing the first 250 iterations. The initial base and spike angle pair is indicated with a green X.

The WE conformational ensemble successfully produces additional conformations not observed in standard MD. To illustrate this point, the base/spike region observed in the apo WE simulations is compared to standard MD in Figure 5a (both a collective 6 μs, with the first 250 iterations of WE simulation and the first 10 ns of standard MD data removed) where, as in Figure 2b, the standard MD results are represented via a boundary. Again, similar results are obtained for the AT-130 WE simulations, i.e., the WE results explore regions outside those sampled in standard MD (Figure S21a), while in contrast, the GLS4-bound tetramers in the WE simulations, relative to the standard MD results, sample a comparable region of the base/spike angle space (Figure S21c).

**Figure 5:**
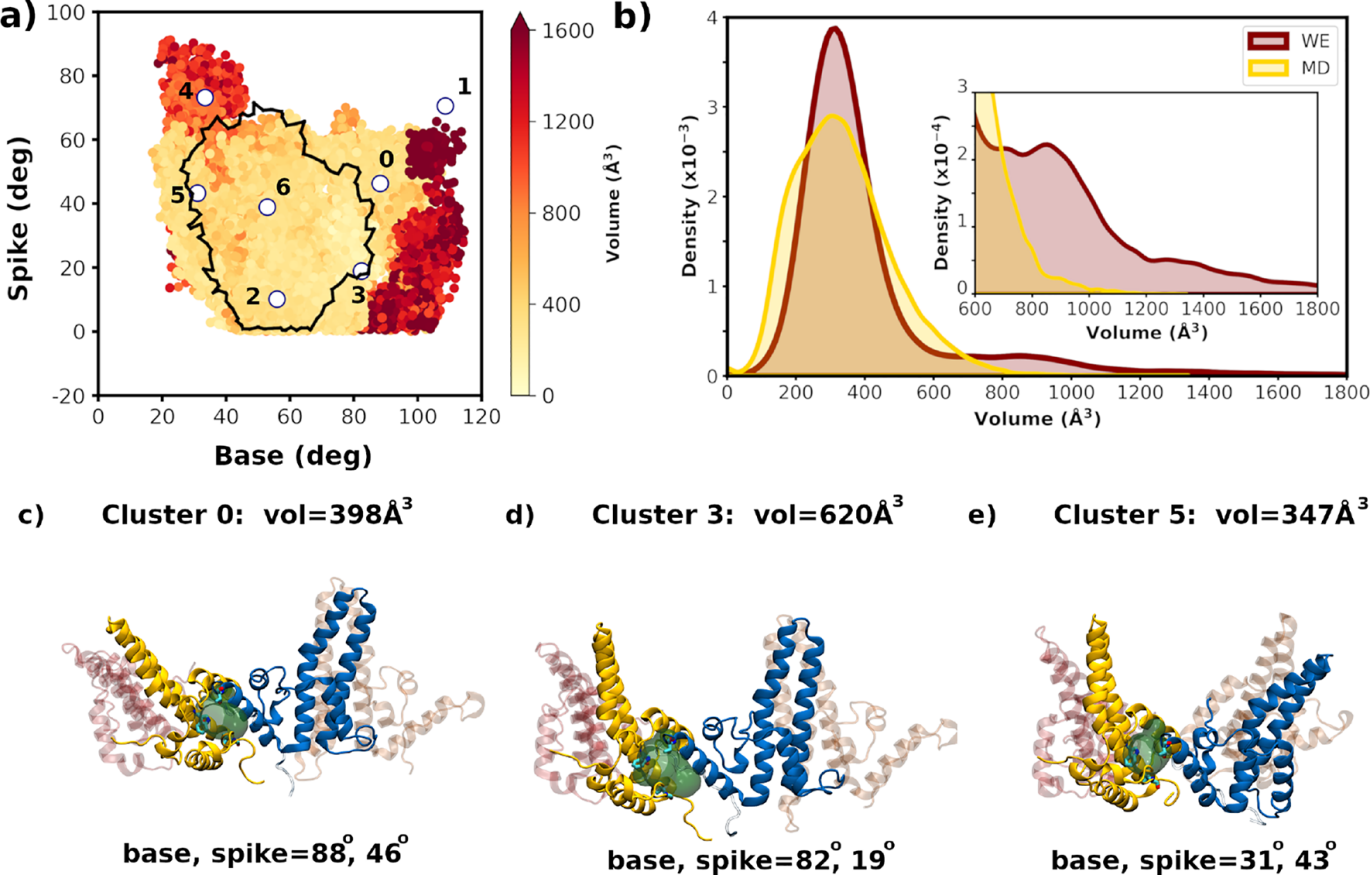
WE samples a wider distribution of base and spike angles and larger ligand binding site volumes. (a) Scatter plot of the base- and spike-angle progress coordinates colored by CAM binding pocket volume. For clarity, the standard MD data is rendered as a boundary (black line). Cluster centers are indicated with white circles and are numbered. (b) Distribution of the CAM pocket volumes for the WE (dark red) and standard MD (gold) simulations. See the inset for an expanded view of the larger volume range. (c–e) Representative tetramer structures from selected clusters showing the Epock pocket volume, along with their base and spike angles. W102, T128, and L140 are rendered in cyan licorice, and the volume is rendered as a dark green surface.

### Apo WE Simulations Explore Enlarged Ligand Binding Sites

Holo structures are often preferred to their apo counterparts for drug discovery purposes as their ligand binding sites are already well-formed,^78^ although such structures may be unavailable for the protein of interest. In such cases, methods to address this shortcoming include template-based approaches to binding site refinement,^79^ metadynamics to explore an enhanced sampling of the binding site,^65^ or induced-fit procedures.^80^ However, WE-based simulations are an attractive alternative to these approaches since the underlying dynamics remains unbiased. In fact, Xu et al.^45^ and Hellemann and Durrant^46^ have applied WE-based simulations to explore ligand binding, with the former focused on the properties of the ligand binding site and the latter demonstrating that including protein flexibility improved ligand binding site exploration.

We have focused on allosteric modulation of the HBV tetramer employing previously determined, mechanistically relevant progress coordinates and have illustrated a wider sampling of the base and spike angle landscape. It is of particular interest to evaluate the ability of these novel WE-derived apo conformations to successfully bind known CAMs. Here we estimate the CAM pocket volumes for the WE-generated apo conformations using Epock.^64^ In Figure 5a, the unweighted WE data is colored by pocket volume; it is noteworthy that the larger volumes correspond to the more extreme values of the progress coordinates, whereas standard MD produces few, if any, conformations with larger volumes. Figure 5b displays the CAM pocket volume distribution for the WE replicas compared with pocket volumes from the standard MD simulations. The distributions are centered about a volume of ∼350 Å^3^ for both the WE and standard MD runs. Although structures with significantly larger than average pocket volumes make up a small fraction of the total for both approaches (Figure 5b), WE simulations generate more large pocket conformations than standard MD (Figure 5b, inset). These structures are likely to be amenable to docking larger CAMs than those with an average ligand binding site volume. We emphasize that the progress coordinates currently used do not explicitly include information concerning the ligand binding site; larger binding site volumes are naturally produced in the course of sampling the functionally relevant base and spike angles. Given that the number of conformations needed to generate a successful ensemble docking protocol can be quite large,^81^ our results suggest that the use of WE simulations will enhance the success rate in ensemble docking efforts. In addition to exploring the CAM binding pocket volume in the apo simulations, the AT-130 and GLS4 simulations were also analyzed (Figure S21b,d). As expected the presence of the ligand reduces the variation in the binding site volumes for both AT-130 as well as GLS4.

Clustering is often employed in ensemble docking in order to extract a small representative subset of protein conformations and is performed on global protein RMSD^23^ or, alternatively, RMSD of the ligand binding site residues directly.^82^ In the present case, although the binding pocket volume is required to be of sufficient size to bind a ligand, it is the functional, i.e., allosteric response of the tetramer, that is thought to drive modulation of HBV capsid assembly. We have performed clustering on the mechanistically relevant degrees of freedom of the HBV tetramer, i.e., the base and spike angles. We note that the present study is meant to illustrate the utility of WE-based simulations to successfully produce conformations, including those not readily sampled in standard MD simulations, for ensemble docking applications and as such is a proof-of-concept study.

Both the WE (unweighted data) and standard MD data were clustered using hierarchical clustering, employing the base and spike angles using ∼50,000 conformations in each case with downsampling performed for the WE data. As discussed by Hellemann and Durrant,^46^ such downsampling may skew the distribution due to the merging and splitting steps in the WE protocol. However, we find that such effects are modest in the base and spike angle distributions used here (Figure S22, where we illustrate the effect of downsampling on the base- and spike-angle distributions as well as on clustering results). The WE data generated seven clusters, whose centers are labeled in Figure 5a, and span the range of base/spike angles. Representative conformations extracted from each cluster produce structures with a range of binding pocket volumes. Several of these conformations are illustrated in Figure 5c-e, labeled with their volumes, base, and spike angles, while structures from all seven clusters, along with the hierarchical dendrogram, are displayed in Figure S23. It is noteworthy that some of the conformations with very extreme values for the base and spike angles also have extremely large volumes (e.g., centers 1 and 4). Note that the center labeled 1 is a single conformation and appears nearly dissociated. Moreover, the locations of these cluster centers are mapped onto the WE-derived free energy landscape (Figure S24), illustrating that several correspond to reasonably rare conformations. While clustering of the apo WE data generated seven clusters, similar clustering of the apo standard MD produced four clusters (Figure S25).

### Apo WE Simulations Provide Suitable Conformations for CAM Docking

In order to validate that the conformations obtained from the apo simulations are conducive to docking, we extracted structures from clusters 0, 2, 3, 5, and 6 of the WE simulations and conformations from the four standard MD clusters. The ligand binding site volumes for the extracted conformations are reported in Table 1. We have chosen CAMs from two mechanistically different classes, i.e., class I misdirectors (GLS4) and class II accelerators (AT-130) for these docking runs. Moreover, GLS4 poses a challenge due to the significantly larger volume required to accommodate it (Figure 2c). The conformations from clusters 1 and 4 from the WE simulations were eliminated from this initial docking calculation as they have extreme values for the base and spike angle pairs and appear nearly dissociated.

**Table 1:** Ligand Binding Pocket Volumes (Å^3^) for Representative Conformations

cluster number	standard MD	cluster number	WE
0	372	0	398
1	496	2	326
2	212	3	620
3	261	5	347
		6	321

Both AT-130 and GLS4 were docked into a volume centered around the CAM binding pocket of the protein using AutoDock Vina^69,70^ with sample conformations shown in Figure 6. For both the GLS4 and AT-130 docking exercises, the ligand is successfully placed in the known CAM ligand binding site, with favorable predicted affinities (<−9 kcal/mol). In the case of GLS4 (Figure 6a–c), while the CAM binding site is identified, the orientation of the ligand is rotated relative to the GLS4-bound structure. Although AutoDock Vina often generates proper ligand-bound poses, especially when a holo structure is employed, it is not uncommon to observe a rotated pose.^69,83^ Similar results are observed for docking AT-130 into the binding site of standard MD based conformations (Figure 6f), i.e., although the binding site is identified, the pose is rotated relative to that observed in the AT-130-bound state (Figure 6d). However, we note that for the WE-derived example given (Figure 6e), although the substituted rings of the amide linkage are rotated, an otherwise reasonable docked pose is generated.

**Figure 6:**
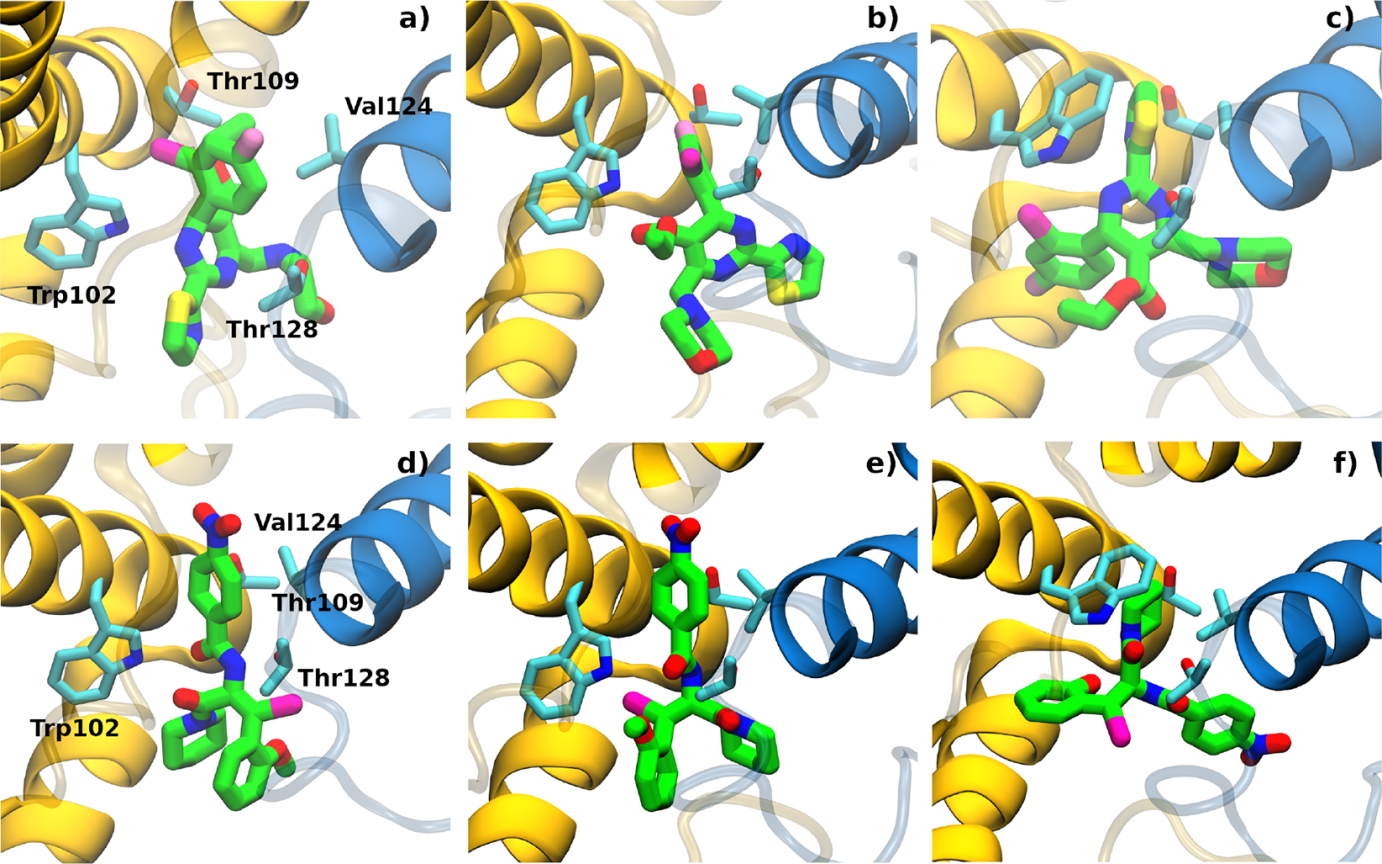
Docking of GLS4 (top row) and AT-130 (bottom row) to the HBV tetramer. (a,d) Initial conformation of the GLS4-bound and AT-130-bound structures. (b,c) Sample conformations with high affinities taken from docking GLS4 into b) WE cluster 3 (docking score −9.7 kcal/mol) and c) into cluster 1 from standard MD (docking score −9.4 kcal/mol). (e,f) Sample conformations with high affinities taken from docking AT-130 into (e) WE cluster 2 (docking score −9.3 kcal/mol) and (f) into cluster 1 from standard MD (docking score −10.1 kcal/mol). Ligands and Cp side chains are rendered in licorice, with carbon, oxygen, nitrogen, sulfur, fluorine, and bromine colored green, red, blue, yellow, pink, and magenta, respectively.

Upon docking AT-130 and GLS4, we find that for both compounds, four out of five conformations derived from the WE data and two out of four from the standard MD data produce results with the ligand placed in the CAM binding site. Coupled with the observation that WE produces an enhanced fraction of large volume conformations (Figure 5b), we conclude that WE offers a reliable way to maximize the number of apo conformations that can be successfully employed.

### Conclusions

CAM efficacy is not only characterized by binding affinity but also by the resulting conformational changes in early assembly intermediates such as the tetramer. Our study demonstrates that standard MD simulations for the apo HBV Cp tetramer rarely sample conformations that display ligand binding site volumes of sufficient size for CAM binding. Furthermore, the standard MD simulations produce conformations that span a restricted region of the functionally relevant HBV tetramer interdimer orientations as characterized by the base and spike angles. However, both of these deficiencies are alleviated by the use of the weighted ensemble approach. Therefore, WE simulations are particularly attractive in drug design programs.

In summary, the main results of the present work include: the use of WE-based simulations not only produces conformations characterized by base and spike angles outside those sampled in standard MD, but also generates an enhanced number of large-volume CAM binding pockets. Furthermore, we show that these WE-produced apo structures can be successfully used in docking of the well studied HBV CAMs of both mechanistically distinct classes, namely AT-130 and GLS4, the latter being a reasonably challenging test due to its size. These results open the way for further virtual screening studies targeting the allosteric modulation of HBV capsid assembly. Additionally, similar to results observed for the apo system, standard and WE-based MD simulations for the AT-130 bound case produce enhanced sampling of the HBV tetramer. In contrast, the exploration of the base and spike angle regions is comparable for the GLS4-bound state, suggesting a ligand-dependent effect. However, additional simulations are needed to confirm this, perhaps with the use of recent enhancements in the WE approach itself as discussed above. Finally, in agreement with earlier studies on protein–ligand thermodynamics,^29,30^ the use of HMR is shown to have only a modest effect on the protein and ligand equilibrium distributions. The present study, along with recent work by Xu et al.^45^ and Hellemann and Durrant,^46^ has established that the WE approach is an effective enhanced sampling method for generating and characterizing ligand binding in the protein conformational landscape and produces viable conformations for further docking studies.

## Supplementary Material



## Data Availability

All standard MD trajectories, as well as scripts and resultant data sets needed for analysis for both standard and WE simulations, are available on Zenodo at 10.5281/zenodo.17381519.

## Notes

Additional analysis of equilibrium simulations, comparison of 2 and 4 fs time step, and progression of the WE simulations (PDF)
