# Supplementary material for: Weighted Ensemble Simulations Reveal Novel Conformations and Modulator Effects in Hepatitis B Virus Capsid Assembly
Source: J Chem Theory Comput. 2025 Nov 25;21(23):12317–27. doi: 10.1021/acs.jctc.5c01197 (PMC12874387; doi:10.1021/acs.jctc.5c01197)
Supplement: Supplementary file 1 [file ct5c01197_si_001.pdf]

Supporting Information: Weighted Ensemble Simulations Reveal Novel Conformations and Modulator Effects in Hepatitis B Virus Capsid Assembly

Diane L. Lynch,[†] Anna Pavlova,[†] Zixing Fan,[‡] and James C. Gumbart[*,†,¶]

†*School of Physics, Georgia Institute of Technology, Atlanta, Georgia, 30332, USA*

‡*Interdisciplinary Bioengineering Graduate Program, Georgia Institute of Technology, Atlanta, Georgia, 30332, USA*

¶*School of Chemistry & Biochemistry, Georgia Institute of Technology, Atlanta, Georgia, 30332, USA*

E-mail: gumbart@physics.gatech.edu

Epock volume encloses AT-130 and GLS4

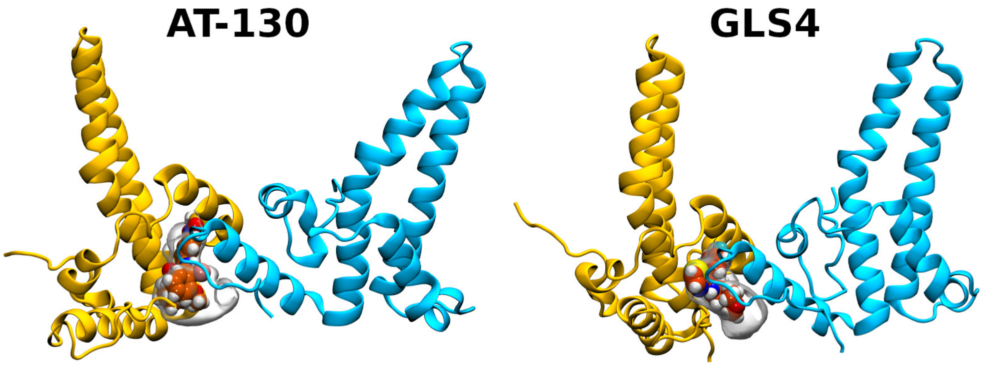

Figure S1: Illustration of the AT-130 and GLS4 CAM binding pockets and the rendered Epock volume. Chain B (gold) and Chain C (blue) are rendered in ribbons, as in Fig. 1. Chains A and D are not displayed. The ligand is displayed with the carbon, oxygen, nitrogen, sulfur, bromine, fluorine, and hydrogen atoms as orange, red, blue, yellow, pink, cyan, and white VDW spheres. The Epock volume is rendered as a transparent white surface, indicating that the region used in the Epock volume calculation is reasonable.

Comparison of 2-fs and 4-fs (HMR) MD Simulations

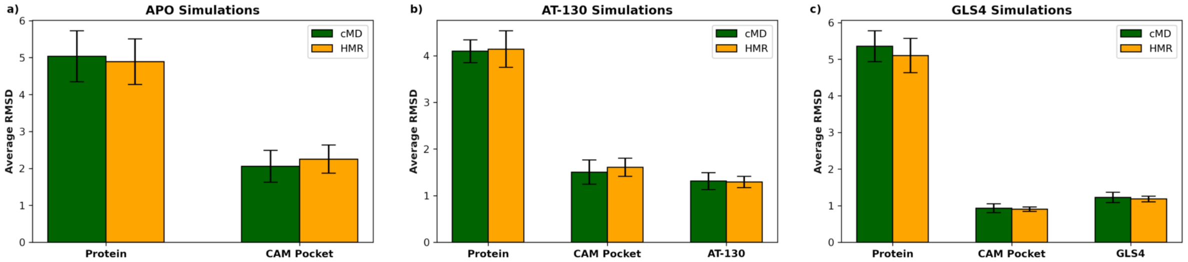

Figure S2: Average root mean square deviation (RMSD) relative to the starting structure, with their 95% confidence intervals. a) Apo simulations with the RMSD aligned and calculated using either all Cα atoms (labeled Protein) or the Cα atoms of the CAM binding pocket residues (defined in the methods section and labeled CAM pocket). b) AT-130 simulations with the protein and CAM binding site calculated as in a). AT-130 RMSDs were calculated using the heavy atoms of the ligand, after alignment of the CAM binding site residue Cα atoms. c) GLS4 simulation results, described as in panel b).

Apo HMR/2-fs Simulations

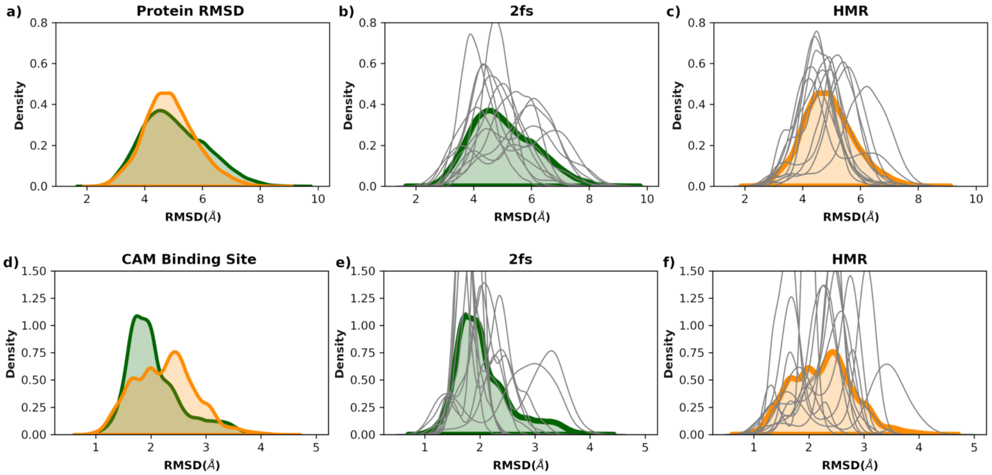

Figure S3: RMSD distributions from the apo simulations. Top row (panels a-c): Both the alignment to the starting structure and the RMSD were calculated using all protein Cα atoms. Bottom row (panels d-f): Alignment and RMSD calculated using the Cα atoms of the CAM binding site residues. Panels a and d report the RMSD distributions for the 12 replicas (combined), comparing the 2-fs simulation results (green) with the HMR results (orange). The middle and right columns are the 2-fs results (panels b and e) and the HMR results (panels c and f), respectively, with the individual replicas plotted separately (grey) and normalized to 1.

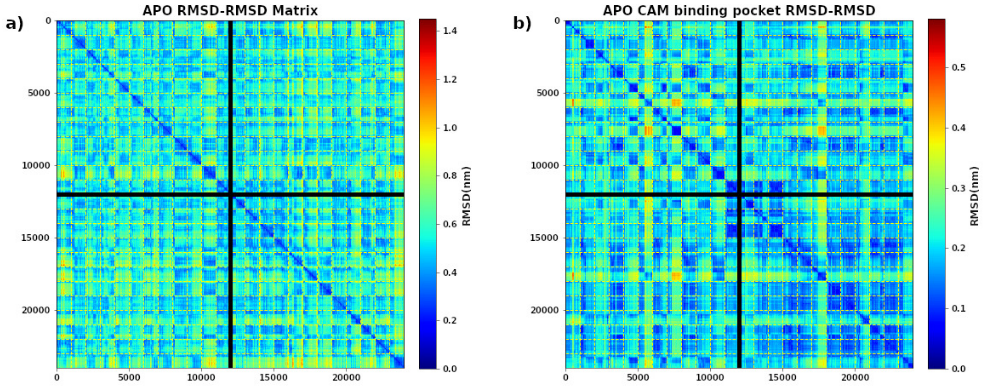

Figure S4: Apo simulation pairwise RMSDs calculated after sequentially concatenating the 12 replicas, with the HMR data followed by the 2-fs data. The RMSD data is computed after alignment using either a) all the protein Cα atoms or b) the Cα atoms of the CAM binding pocket residues. The HMR and 2-fs timestep simulations are separated by the black vertical/horizontal lines with the axes labeling individual conformations. The 2-fs and HMR simulations are sampled at 0.5 ns, resulting in a combined total of 24,000 conformations.

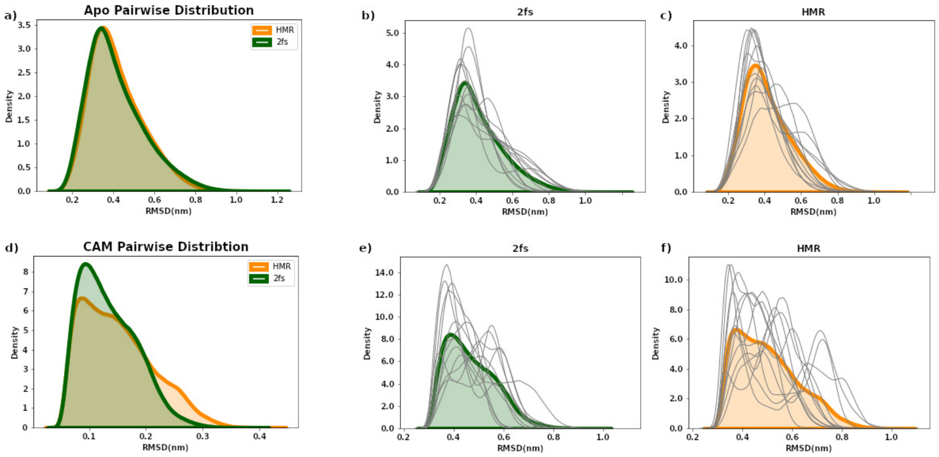

Figure S5: Distribution of pairwise RMSD values from the apo simulations comparing the HMR to the 2-fs timestep results. Top row (panels a-c), RMSDs computed using all protein Cα atoms. Lower row (panels d-f), RMSD results using the CAM Cα atoms of the binding site residues. Panels a and d report the pairwise RMSD distributions for the 12 replicas (combined), with the 2-fs simulation results (green) and the HMR results (orange). The middle and right columns are the 2-fs results (panels b and e) and the HMR results (panels c and f), respectively, with the individual replicas plotted separately (grey) and normalized to 1.

AT-130 HMR/2-fs Simulations

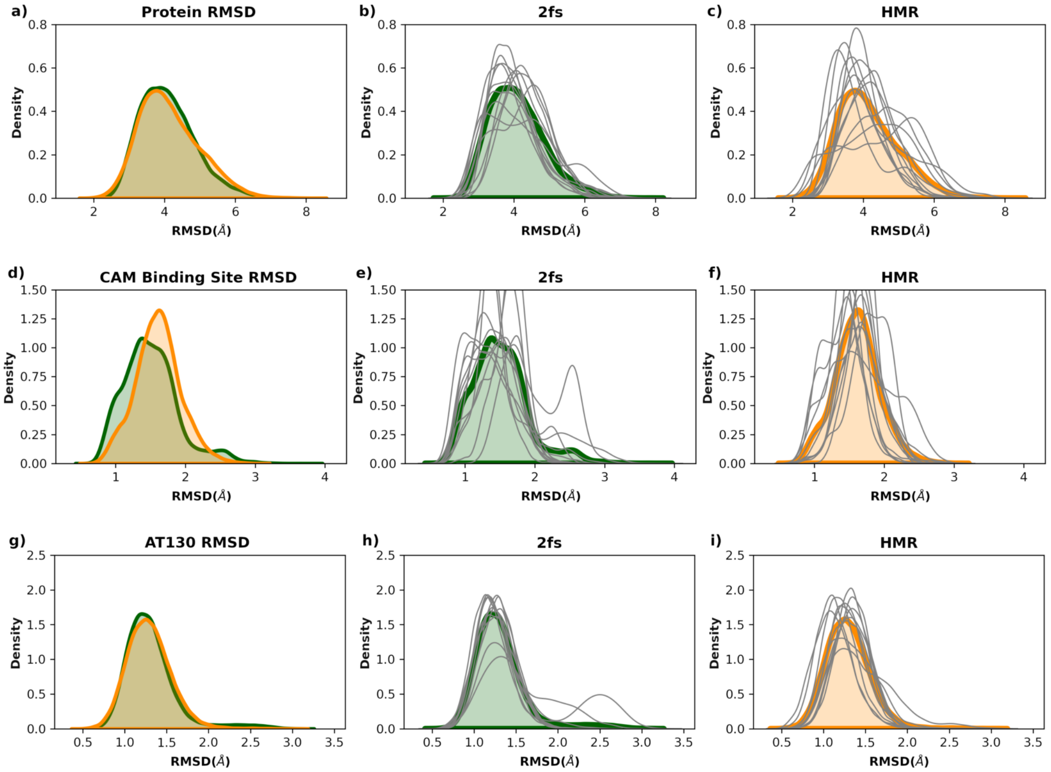

Figure S6: RMSD distributions from the AT-130-bound simulations. Top row (panels a-c): Both the alignment to the starting structure and the RMSD were calculated using all protein Cα atoms. Middle row (panels d-f): Alignment and RMSD calculated using the Cα atoms of the CAM binding site residues. Lower row (panels g-i): RMSD of the AT-130 heavy atoms after alignment using the Cα atoms of the CAM binding site residues. Panels a, d, and g report the RMSD distributions for the 12 replicas (combined), comparing the 2-fs simulation results (green) with the HMR results (orange). The middle and right columns are the 2-fs results (panels b, e, and h) and the HMR results (panels c, f, and i), respectively, with the individual replicas plotted separately (grey) and normalized to 1.

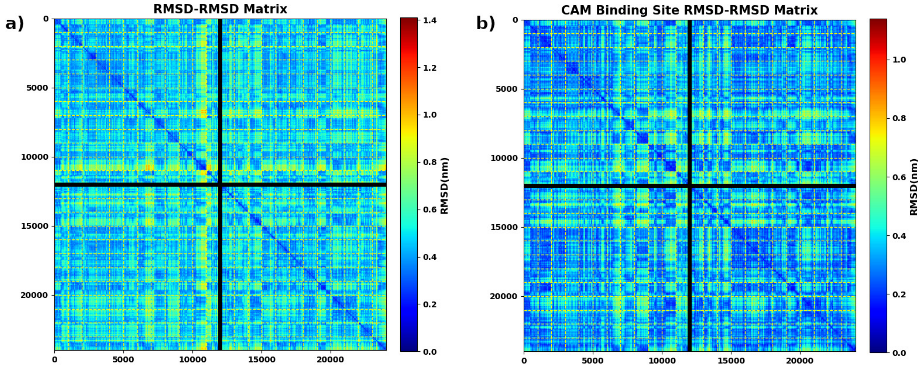

Figure S7: AT-130-bound simulation pairwise RMSDs calculated after sequentially concatenating the 12 replicas, with the HMR data followed by the 2-fs data. The RMSD data is computed after alignment using either a) all the protein Cα atoms or b) the Cα atoms of the CAM binding pocket residues. The HMR and 2-fs timestep simulations are separated by the black vertical/horizontal lines with the axes labeling individual conformations. The 2-fs and HMR simulations are sampled at 0.5 ns, resulting in a combined total of 24,000 conformations.

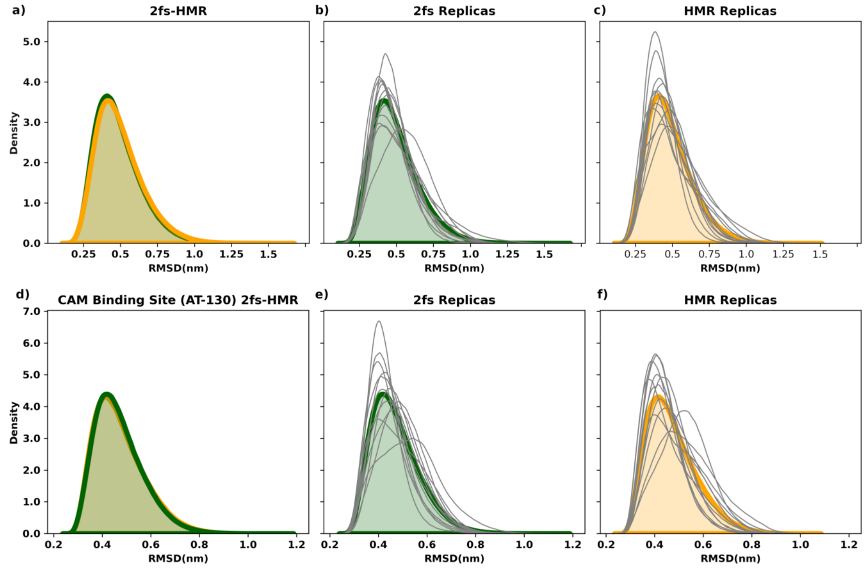

Figure S8: Distribution of pairwise RMSD values from the AT-130-bound simulations comparing the HMR to the 2-fs timestep results. Top row (panels a-c), RMSD computed using all protein Cα atoms. Lower row (panels d-f), analogous results using the CAM Cα atoms of the binding site residues. Panels a and d report the pairwise RMSD distributions for the 12 replicas (combined), with the 2-fs simulation results (green) and the HMR results (orange). The middle and right columns are the 2-fs results (panels b and e) and the HMR results (panels c and f), respectively, with the individual replicas plotted separately (grey) and normalized to 1.

GLS4 HMR/2-fs MD Simulations

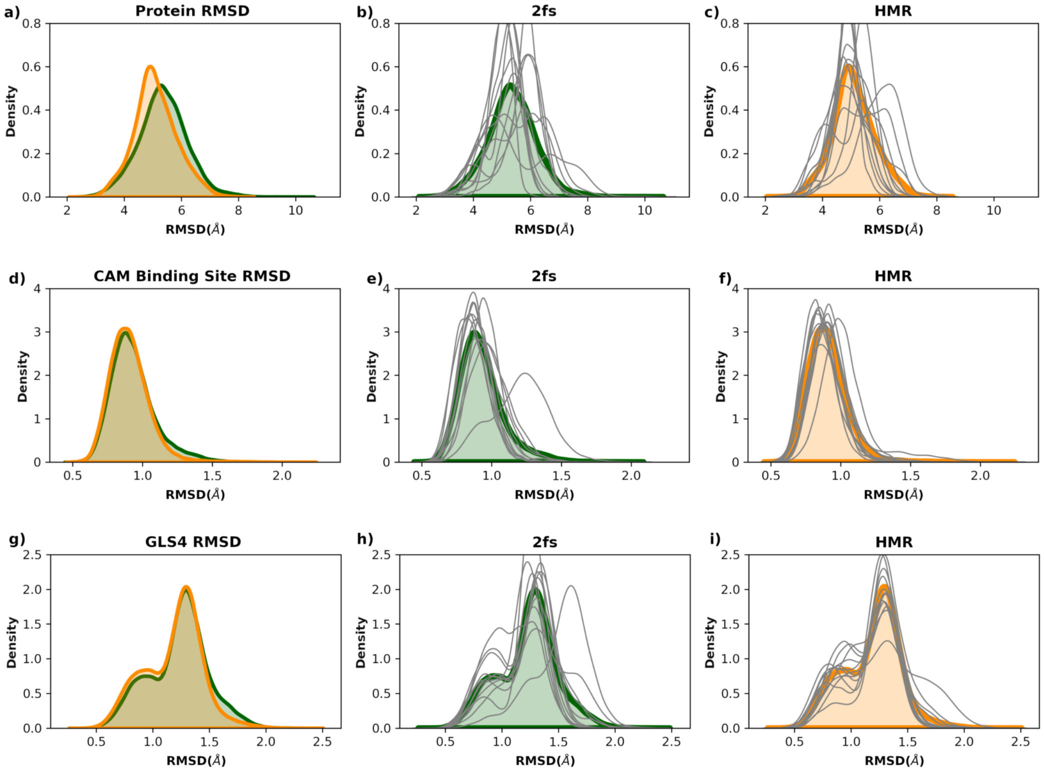

Figure S9: RMSD distributions from the GLS4-bound simulations. Top row (panels a-c): Both the alignment to the starting structure and the RMSD were calculated using all protein Cα atoms. Middle row (panels d-f): Alignment and RMSD calculated using the Cα atoms of the CAM binding site residues. Lower row (panels g-i): RMSD of the GLS4 heavy atoms after alignment using the Cα atoms of the CAM binding site residues. Panels a, d, and g report the RMSD distributions for the 12 replicas (combined), comparing the 2-fs simulation results (green) with the HMR results (orange). The middle and right columns are the 2-fs results (panels b, e, and h) and the HMR results (panels c, f, and i), respectively, with the individual replicas plotted separately (grey) and normalized to 1.

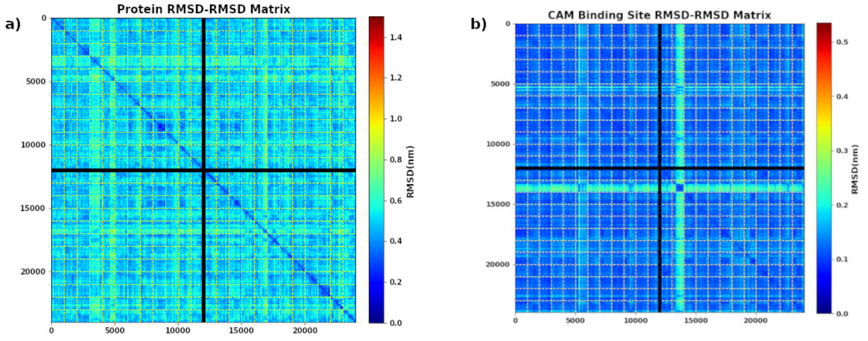

Figure S10: GLS4-bound simulation pairwise RMSDs calculated after sequentially concatenating the 12 replicas, with the HMR data followed by the 2-fs data. The RMSD data is computed after alignment using either a) all the protein Cα atoms or b) the Cα atoms of the CAM binding pocket residues. The HMR and 2-fs timestep simulations are separated by the black vertical/horizontal lines with the axes labeling individual conformations. The 2-fs and HMR simulations are sampled at 0.5 ns, resulting in a combined total of 24,000 conformations.

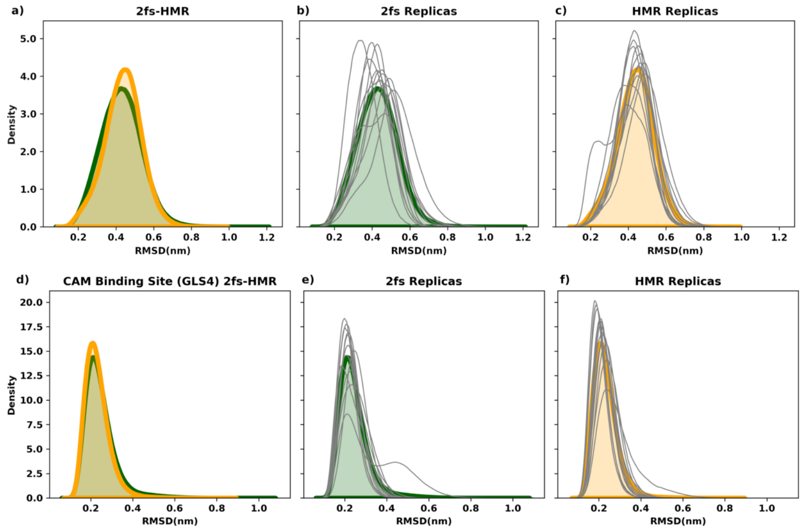

Figure S11: Distribution of pairwise RMSD values from the GLS4-bound simulations comparing the HMR to the 2-fs timestep results. Top row (panels a-c), RMSD computed using all protein Cα atoms. Lower row (panels d-f), analogous results using the CAM Cα atoms of the binding site residues. Panels a and d report the pairwise RMSD distributions for the 12 replicas (combined), with the 2-fs simulation results (green) and the HMR results (orange). The middle and right columns are the 2-fs results (panels b and e) and the HMR results (panels c and f), respectively, with the individual replicas plotted separately (grey) and normalized to 1.

Comparing 2-fs and HMR results for base and spike angles

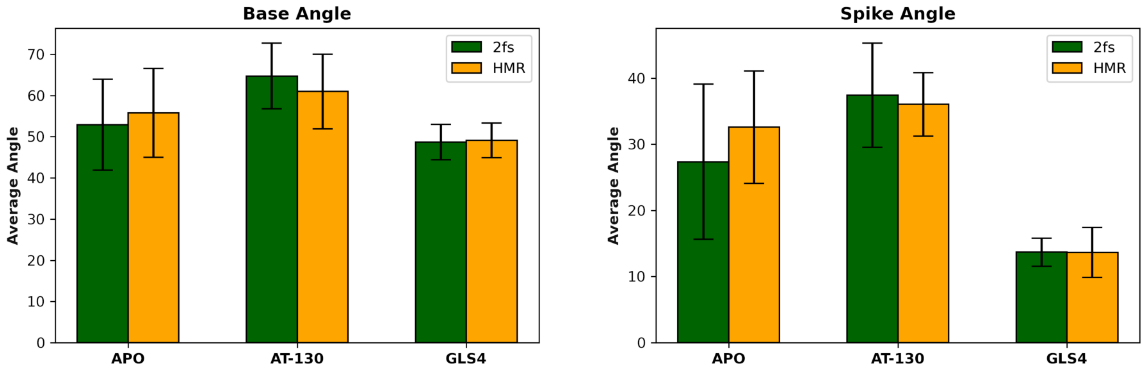

Figure S12: Average base and spike angles with the 95% confidence interval computed for the apo, as well as the AT-130- and GLS4-bound simulations. No statistical difference is seen with the use of hydrogen mass repartitioning.

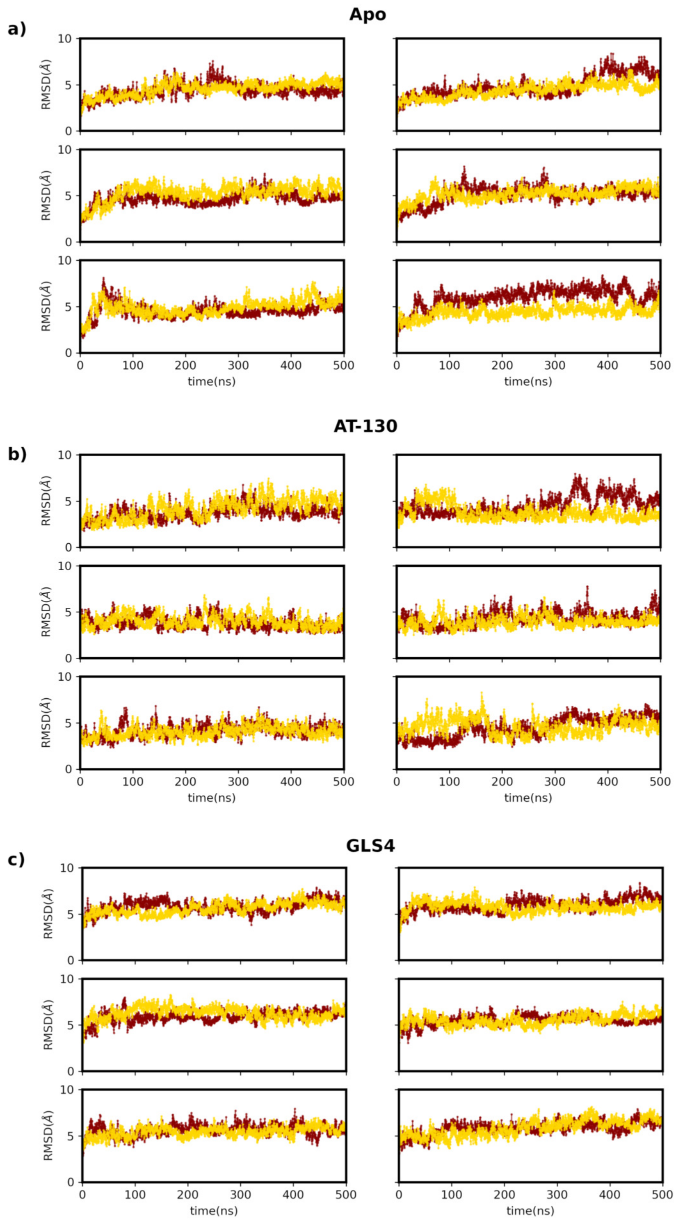

Figure S13: Timeseries of the protein RMSD, using all the Cα atoms for alignment and RMSD measurement, for each of the 12 replicas. a) Apo, b) AT-130, and c) GLS4 simulations. Individual replicas are distinguished by color.

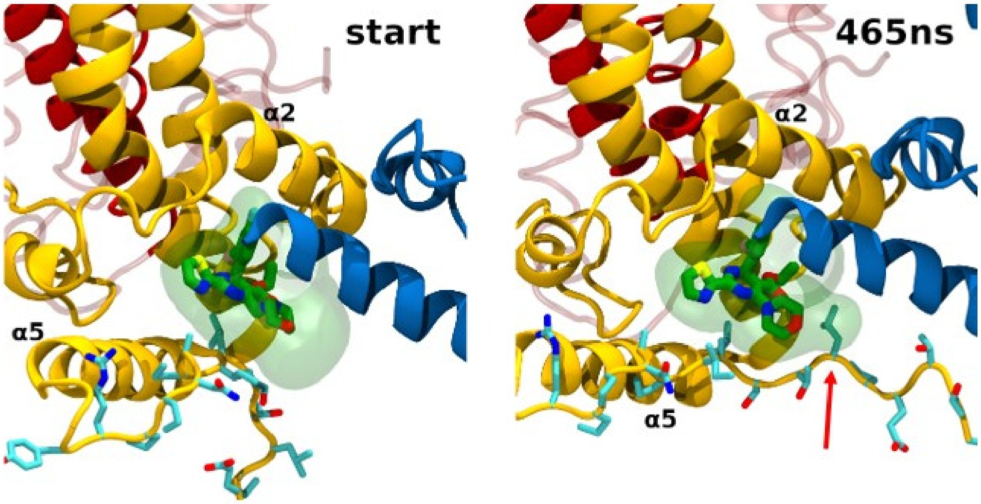

Figure S14: GLS4 Tail Conformations. The left panel is a snapshot of the starting conformation and the right panel is taken at 465 ns. The C-terminal tail is rendered licorice and occasionally wraps around and occludes available space near the ligand reducing the volume (area indicated by red arrow). Ligands rendered in a thicker licorice with carbon, oxygen, nitrogen, and sulfur colored green, red, blue, and yellow respectively.

WE simulations

Apo

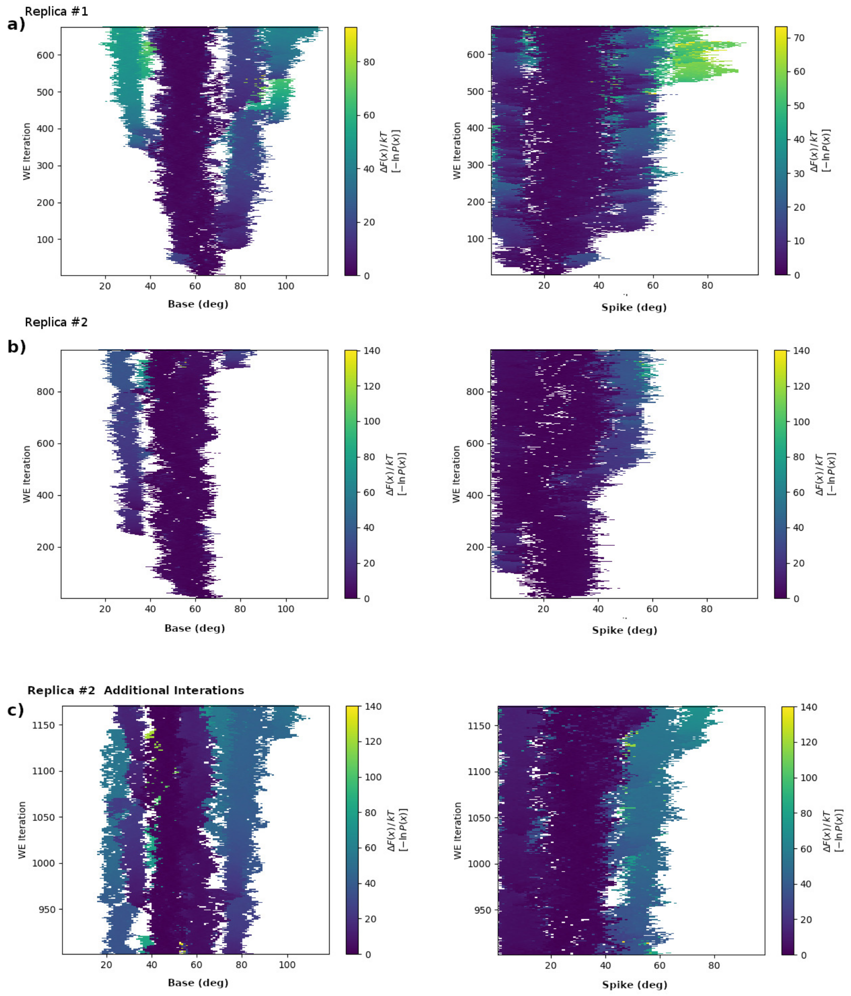

Figure S15: Evolution of the progress coordinates. a)and b) Evolution for the two independent apo replicas, with the base angle in the left column and the spike angle in the right column and each replica run to 3 μs. c) Extending the second replica from 960 to 1170 iterations reveals that it displays a further exploration of the base and spike progress coordinates.

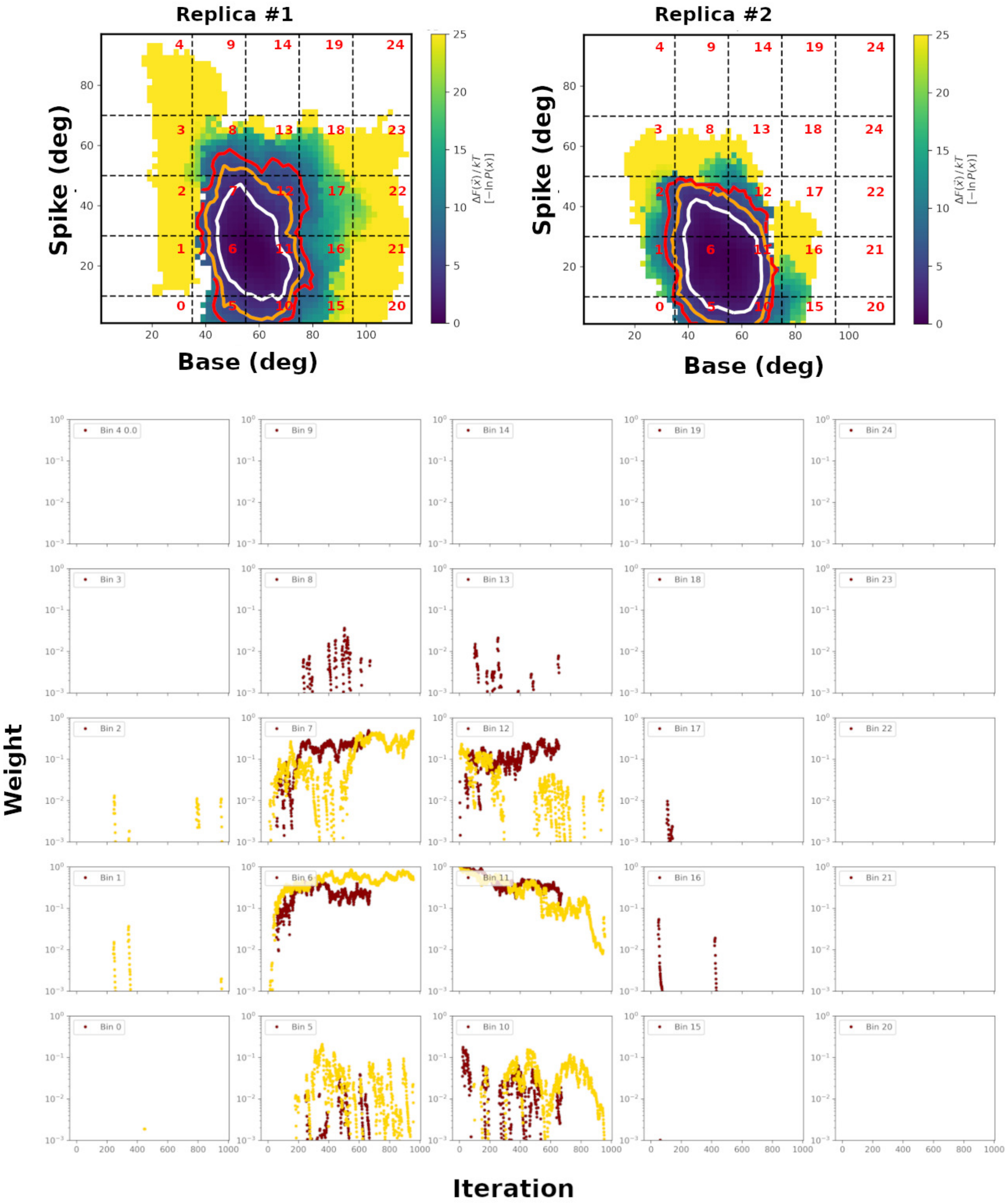

Figure S16: 2-D progress coordinate landscape for apo simulations: a) replica 1 and b) replica 2. The bins are numerically labeled from 0 to 24 for the 2-D base and spike progress coordinates, with the bin boundaries indicated by black dashed lines and contours plotted at $2.5kT$(white), $5.0kT$(orange), and $7.5kT$(red). c) Statistical weights as a function of WE iteration for each bin, numbered as in panels a) and b), with replica 1 colored red and replica 2 colored gold.

AT-130

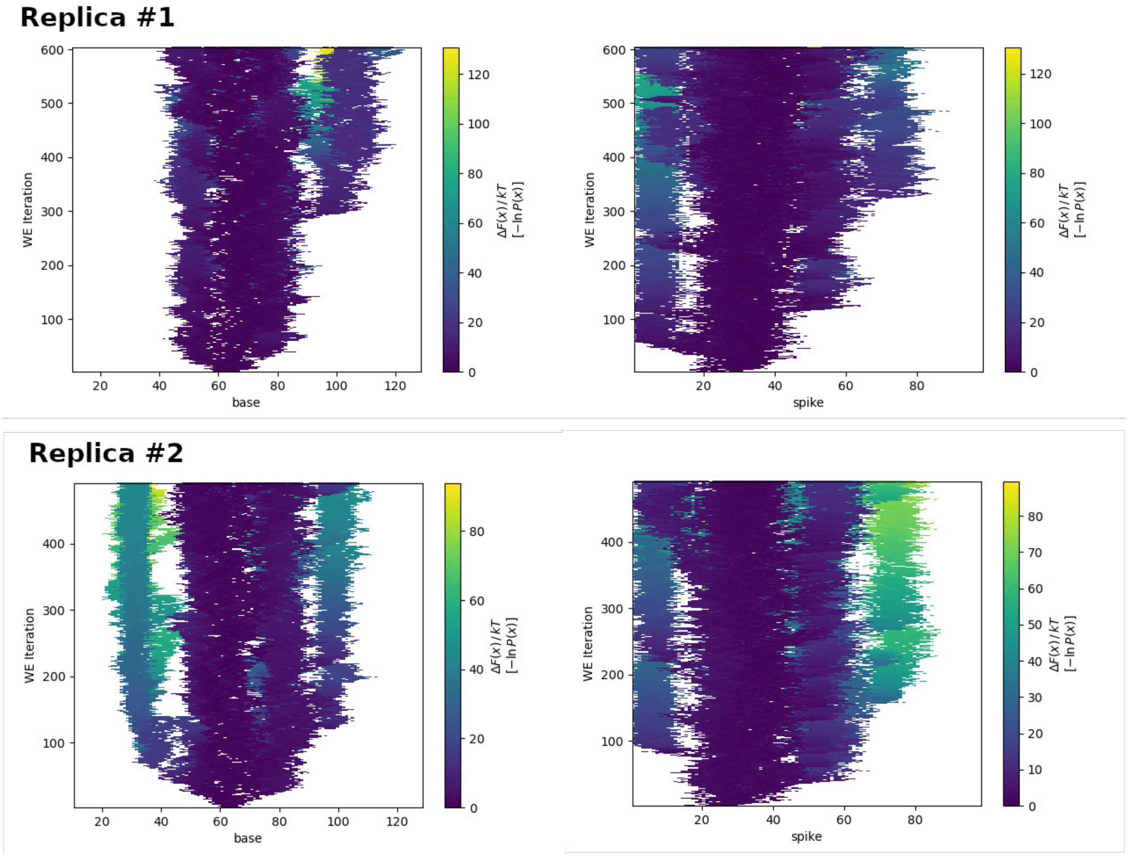

Figure S17: Evolution of the progress coordinates for the two independent AT-130 replicas, with the base angle in the left column and the spike angle in the right column and each replica run to 3 μs.

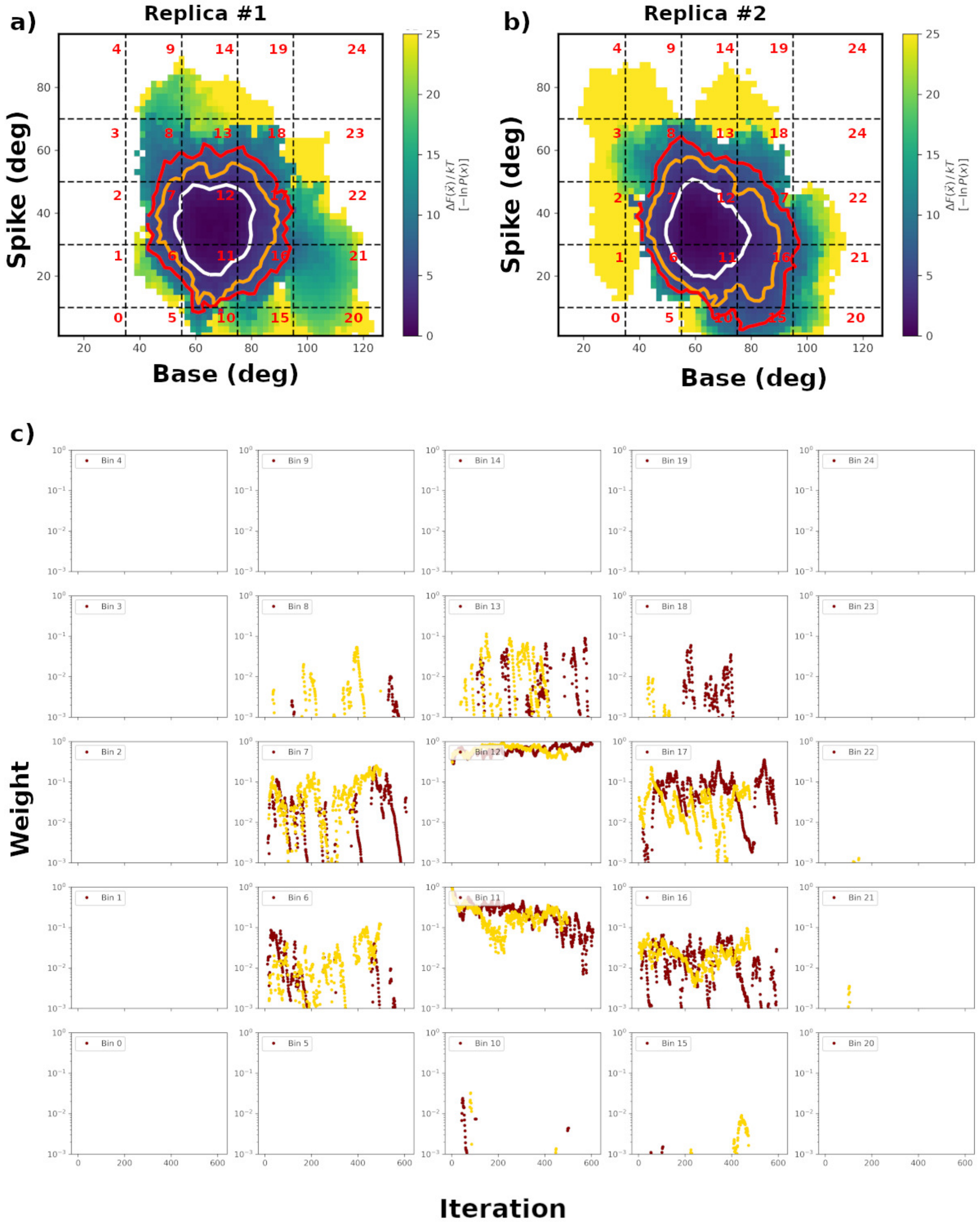

Figure S18: 2-D progress coordinate landscape for AT-130-bound simulations: a) replica 1 and b) replica 2. The bins are numerically labeled from 0 to 24 for the 2-D base and spike progress coordinates, with the bin boundaries indicated by black dashed lines and contours plotted at $2.5kT$(white), $5.0kT$(orange), and $7.5kT$(red). c) Statistical weights as a function of WE iteration for each bin, numbered as in panels a) and b), with replica 1 colored red and replica 2 colored gold.

GLS4

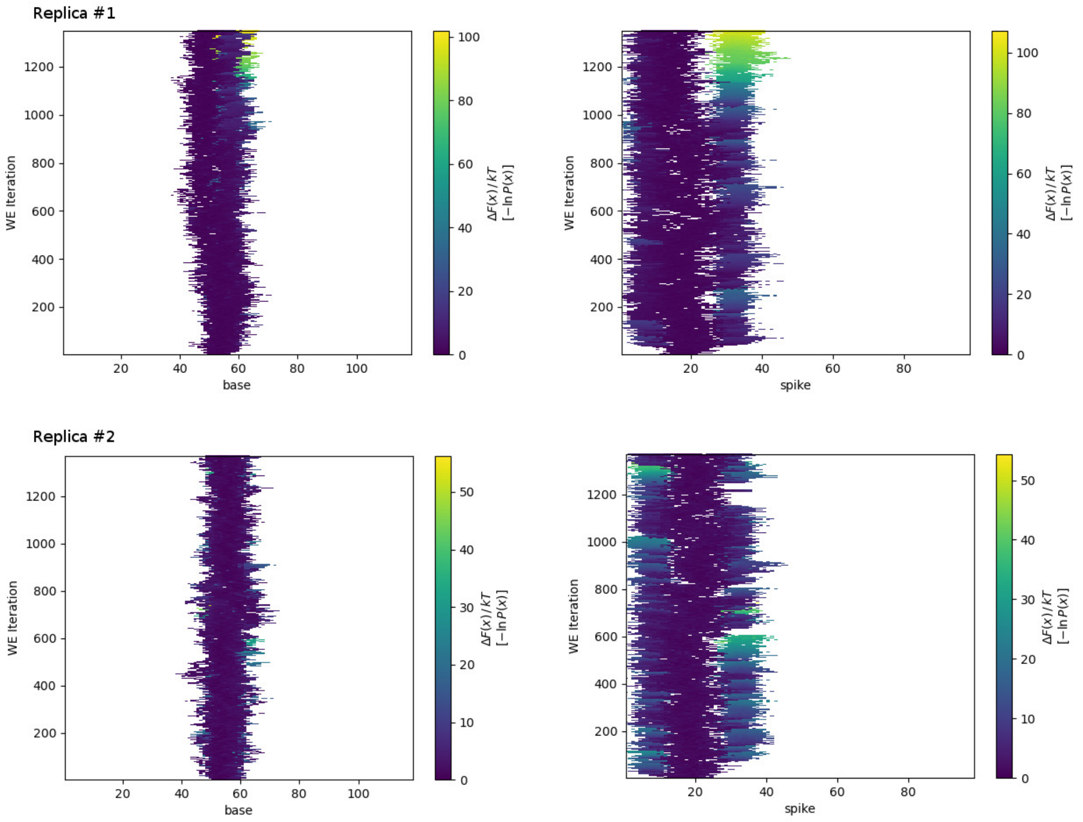

Figure S19: Evolution of the progress coordinates for the two independent GLS4 replicas, with the base angle in the left column and the spike angle in the right column and each replica run to 3 μs.

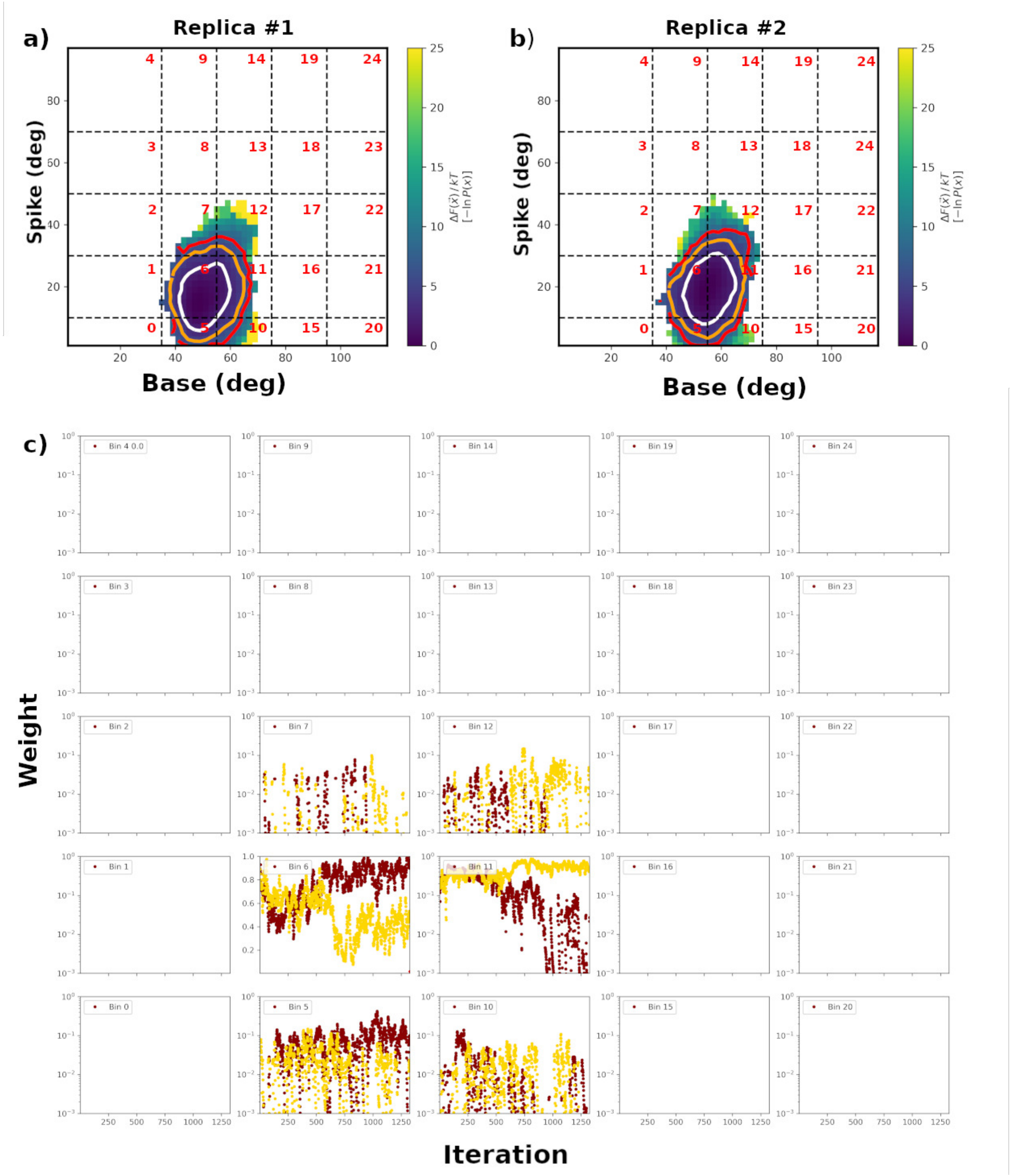

Figure S20: 2-D progress coordinate landscape for GLS4-bound simulations: a) replica 1 and b) replica 2. The bins are numerically labeled from 0 to 24 for the 2-D base and spike progress coordinates, with the bin boundaries indicated by black dashed lines and contours plotted at $2.5kT$(white), $5.0kT$(orange), and $7.5kT$(red). c) Statistical weights as a function of WE iteration for each bin, numbered as in panels a) and b), with replica 1 colored red and replica 2 colored gold.

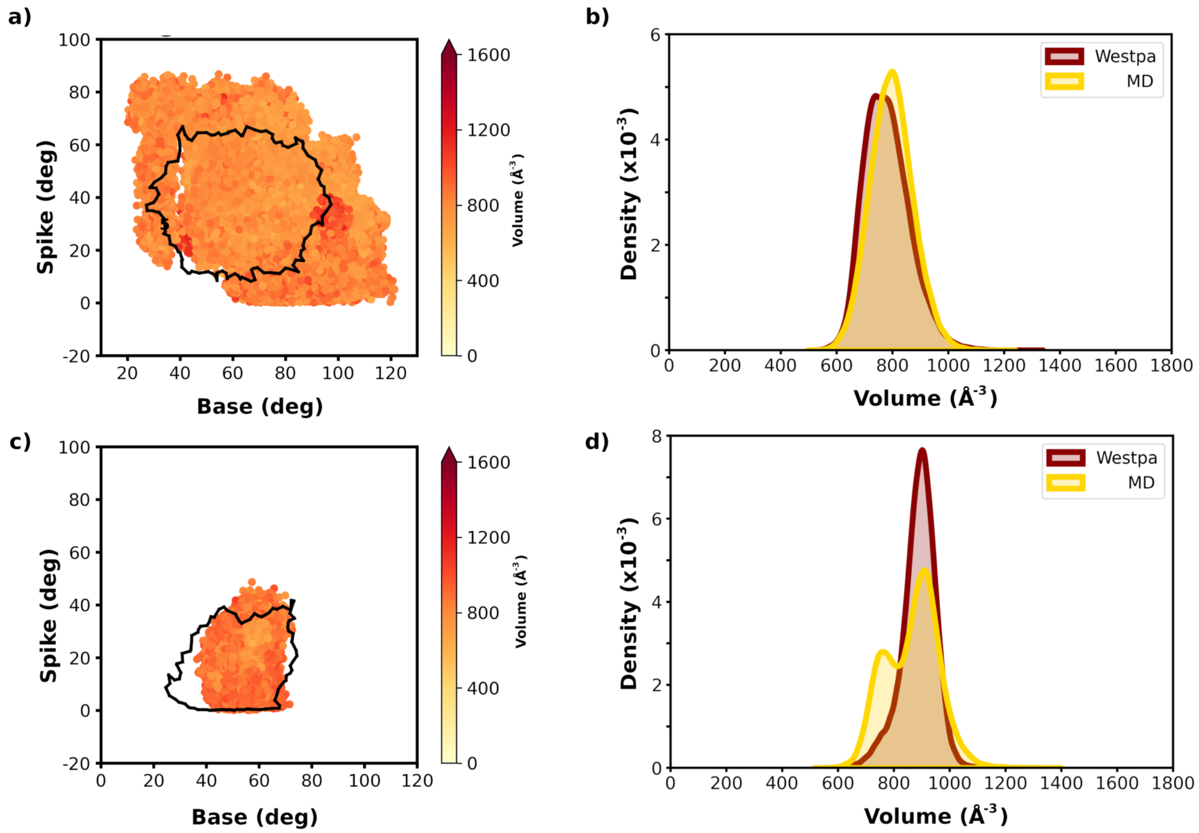

Figure S21: Base and spike angle scatter plots, as well as CAM binding pocket volume distributions, for the AT-130 and GLS4 WE and standard MD simulations. Scatter plots of the base and spike angle progress coordinates (unweighted) for the AT-130 (panel a) and the GLS4 (panel c) simulations. Collective data is reported, with the first 250 iterations of WE simulation removed and the first 10 ns of standard MD data removed. For clarity, the standard MD data is rendered as a boundary (black line) which outlines the region sampled by the 12 replicas, while the WE data is a scatter plot colored by the CAM pocket volume, calculated using Epock. Panels b) and d) display distributions of the WE and standard MD-derived ligand binding pocket volumes for the AT-130 (panel b) and GLS4 (panel d) simulations.

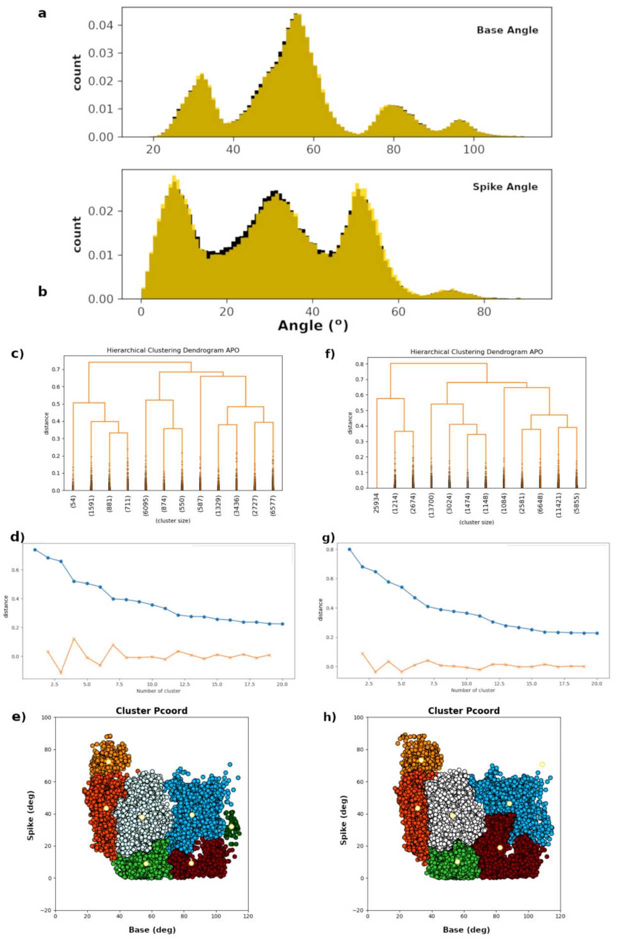

Figure S22: Base (a) and spike (b) histograms of WE (unweighted) downsampled data. The black histograms (generated with the Matplotlib hist function) are the full data set, while the gold is downsampled, with every second data point retained. c-h) Effect on clustering upon increasing the number of conformations with panels on left (c, d, and e) using ∼25000 conformations, while those on the right (f, g, and h) include ∼50000. (c and f) Clustering dendrograms with the distances in units of radians. (d and g) Distance (blue data) and curvature (orange data) as the clusters are merged. Seven clusters are chosen since the local curvature is a maximum for seven clusters. (e and h) display the base/spike 2-D scatter plot colored by cluster number illustrating the slight shifting of clusters as more conformations are added.

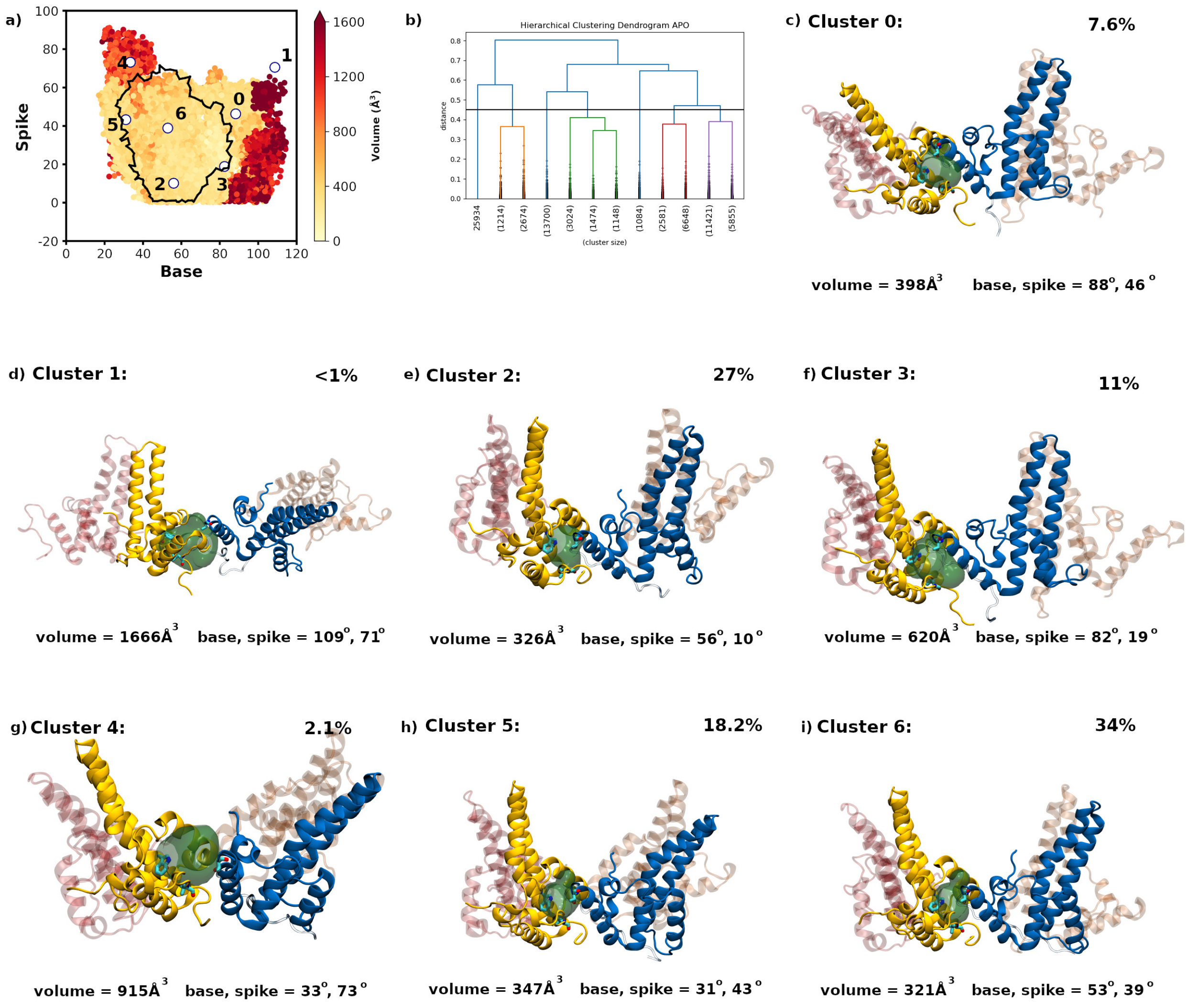

Figure S23: Clustering of the apo WE simulations. a) Scatter plot of the base and spike angle progress coordinates (unweighted; see Fig. 5a in main text). b) Hierarchical dendrogram for the average linkage clustering. Solid line represents the cut-off for the seven clusters. Panels c) - i) are representative conformations from each cluster, taken as the structure with base/spike angle pair closest to the center. The tetramer structures are rendered as chain A (red), chain B (gold), chain C (blue), and chain D (orange), with the Epock volumes illustrated with a dark green surface. W102, T128, and L140 sidechains are displayed in licorice with carbon/oxygen atoms colored cyan/red, respectively. The volume, base, and spike angles are reported below each structure, with the percentage of total conformations contributed by each cluster indicated.

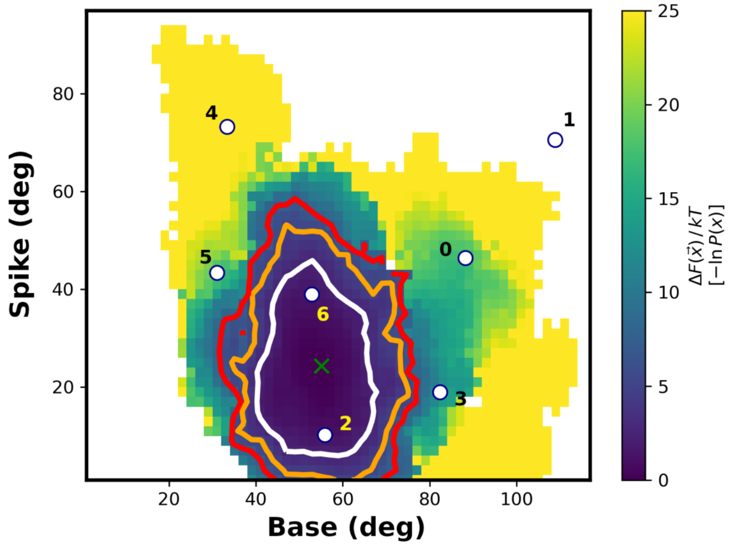

Figure S24: 2-D landscape of Apo simulations after combining the two WE replicas and indicating the locations of the cluster centers used in the docking. The cluster centers are indicated with a white circle and numbered as in Fig. 5a of the main text. The green X indicates the base and spike angles of the starting structure.

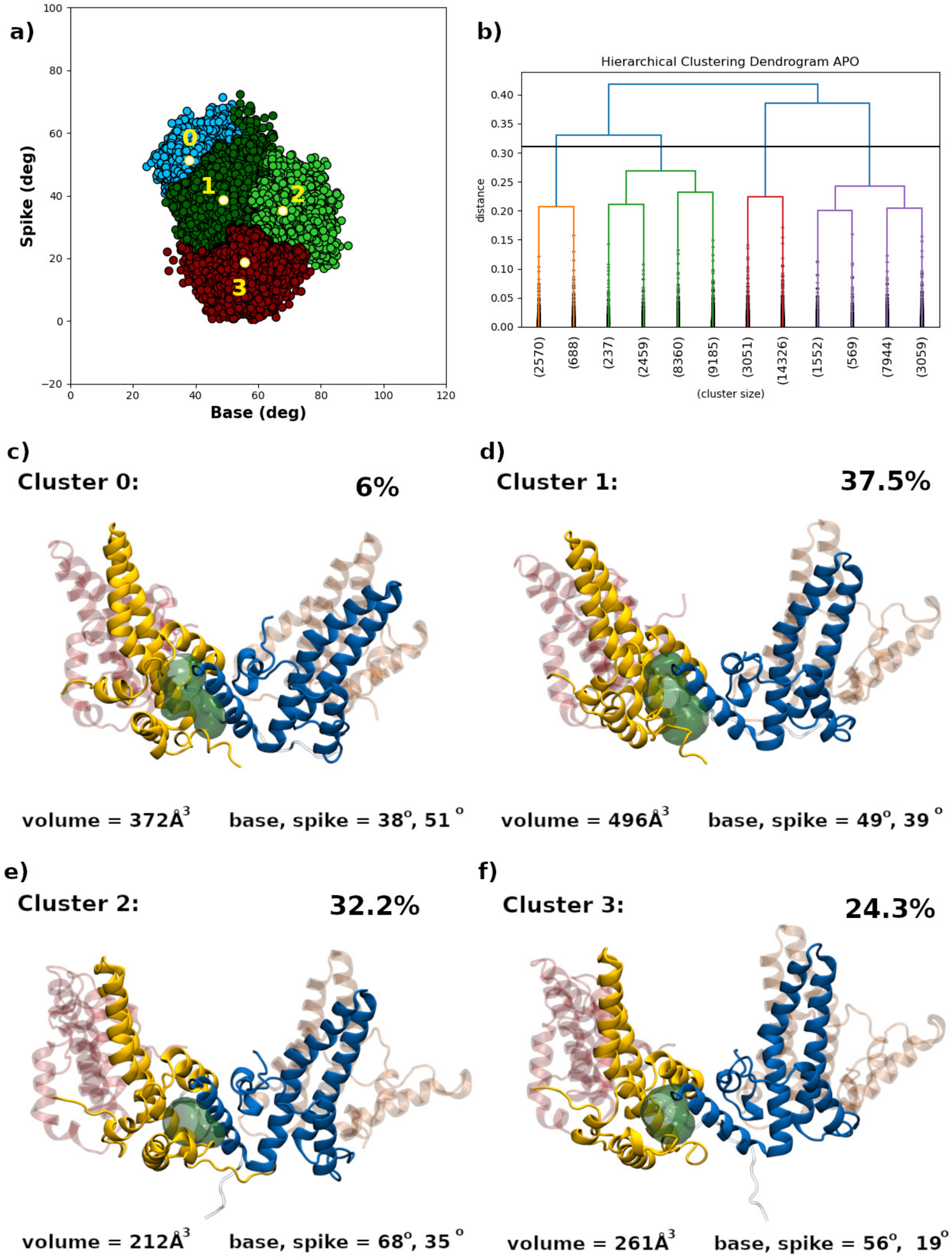

Figure S25: Clustering of the apo standard MD simulations. a) Scatter plot of the base/spike angles colored by cluster membership. The conformation in each cluster closest to the cluster center is indicated with a white circle. b) Hierarchical dendrogram for the average euclidean clustering of the standard apo MD simulations, with initial 10 ns removed. Solid line represents the cut-off for four clusters. Panels c) - f) representative conformations from each cluster, taken as the structure with base/spike angle pair closest to the center. The structures are rendered as chain A (red), chain B (gold), chain C (blue), and chain D (orange), with the Epock volumes illustrated with a green surface. The volume, base, and spike angles are reported below each structure, with the percentage of total conformations contributed by each cluster indicated.
